# Cracking the wall: the fungal cell wall assembly protein ECM33 is a promising molecular target in powdery mildew fungi

**DOI:** 10.1093/hr/uhag101

**Published:** 2026-03-13

**Authors:** Isabel Padilla-Roji, Alejandro Jiménez-Sánchez, Sara Yugueros, Hugo Mélida, Álvaro Polonio, Dolores Fernández Ortuño, Alejandro Pérez-García

**Affiliations:** Departamento de Microbiología, Facultad de Ciencias, Universidad de Málaga, Málaga, Spain; Instituto de Hortofruticultura Subtropical y Mediterránea ‘La Mayora’, Universidad de Málaga, Consejo Superior de Investigaciones Científicas (IHSM−UMA−CSIC), Málaga, Spain; Departamento de Microbiología, Facultad de Ciencias, Universidad de Málaga, Málaga, Spain; Instituto de Hortofruticultura Subtropical y Mediterránea ‘La Mayora’, Universidad de Málaga, Consejo Superior de Investigaciones Científicas (IHSM−UMA−CSIC), Málaga, Spain; Área de Fisiología Vegetal, Departamento de Ingeniería y Ciencias Agrarias, Universidad de León, León, Spain; Instituto de Biología Molecular, Genómica y Proteómica (INBIOMIC), Universidad de León, León, Spain; Área de Fisiología Vegetal, Departamento de Ingeniería y Ciencias Agrarias, Universidad de León, León, Spain; Instituto de Biología Molecular, Genómica y Proteómica (INBIOMIC), Universidad de León, León, Spain; Departamento de Microbiología, Facultad de Ciencias, Universidad de Málaga, Málaga, Spain; Instituto de Hortofruticultura Subtropical y Mediterránea ‘La Mayora’, Universidad de Málaga, Consejo Superior de Investigaciones Científicas (IHSM−UMA−CSIC), Málaga, Spain; Departamento de Microbiología, Facultad de Ciencias, Universidad de Málaga, Málaga, Spain; Instituto de Hortofruticultura Subtropical y Mediterránea ‘La Mayora’, Universidad de Málaga, Consejo Superior de Investigaciones Científicas (IHSM−UMA−CSIC), Málaga, Spain; Departamento de Microbiología, Facultad de Ciencias, Universidad de Málaga, Málaga, Spain; Instituto de Hortofruticultura Subtropical y Mediterránea ‘La Mayora’, Universidad de Málaga, Consejo Superior de Investigaciones Científicas (IHSM−UMA−CSIC), Málaga, Spain

## Abstract

Cucurbit powdery mildew, predominantly caused by *Podosphaera xanthii*, poses a major threat to global cucurbit production due to the pathogen’s rapid adaptability and resistance to conventional fungicides. This growing challenge highlights the urgent need for alternative, sustainable disease management strategies. As the primary interface between the fungus, host plant, and environment, the fungal cell wall emerges as a strategic target for development of innovative control approaches. This study focuses on ECM33, a glycophosphatidylinositol (GPI)-anchored protein believed to play a role in cell wall architecture and integrity, although its specific biochemical function remains undefined. *In silico* structural modeling revealed that PxECM33 resembles leucine-rich repeat proteins and contains potential carbohydrate-binding motifs. Recombinant PxECM33 exhibited binding affinity for chitin, β-glucans, and mannans, the main glycosidic components of the *P. xanthii* cell wall, supporting these structural predictions. Molecular docking analyses uncovered distinct ligand-specific interactions with these carbohydrates, further implicating PxECM33 in cell wall dynamics. Silencing *PxECM33* via RNA interference significantly impaired fungal growth and caused pronounced cell wall disorganization. Notably, dual silencing with the melon immune receptor gene *CmCERK1* mitigated these defects, suggesting that PxECM33 may function in masking immunogenic oligosaccharides to evade host detection. Furthermore, spray-induced gene silencing (SIGS) targeting *PxECM33* effectively reduced disease symptoms in melon plants, highlighting its potential as a sustainable and nontoxic biocontrol strategy. Given the high-sequence conservation of *ECM33* among ascomycete fungi, these findings support its candidacy as a broad-spectrum molecular target for managing powdery mildew in cucurbits and potentially other crops.

## Introduction

Powdery mildews are among the most prevalent and recognizable plant diseases, occurring frequently across a wide range of species. These diseases are caused by powdery mildew fungi (Erysiphales), obligate biotrophic parasites that have a significant impact on agriculture, substantially decreasing crop yields across a wide spectrum of plants, including barley, wheat, grapes, cucurbits, tomatoes, fruits, and ornamental plants, whether in the field or greenhouse conditions. Among these crops, cucurbits are particularly susceptible to powdery mildew, with *Podosphaera xanthii* being the main cause behind this disease, wreaking havoc on crop productivity [1, 2]. To manage cucurbit powdery mildew, growers primarily rely on chemical control and the use of resistant cultivars [3]. However, the rise of new physiological races [1] and isolates resistant to commonly used anti-powdery mildew fungicides [4] has made disease control increasingly challenging. Consequently, there is an urgent need for innovative disease management strategies to keep this persistent plague in check.

The fungal cell wall is a complex structure composed of glucan, chitin, mannans, minor polysaccharides (including rhamnose, galactose, and xylan), and glycoproteins [5, 6]. It is essential for maintaining cell shape and structural integrity, providing mechanical resistance to osmotic pressure, and protecting against toxic compounds [5]. Additionally, the cell wall facilitates interactions with the environment through adhesins and receptors that, upon activation, initiate intricate intracellular signaling cascades [5]. In phytopathogenic fungi, the initiation of the infection frequently begins with cell wall glycoproteins detecting external signaling factor molecules. Beyond supporting growth, the cell wall activates essential intracellular signaling pathways and serves as a primary target for immune recognition in plants [7]. Therefore, a thorough understanding of the fungal cell wall is vital for developing antifungal agents, as it represents a key target for disrupting fungal growth and infection processes [8].

Proteins represent an essential component in the structural makeup of the fungal cell wall. Proteins covalently attached to the cell wall primarily consist of those with conventional signal peptides, often featuring a glycosylphosphatidylinositol (GPI) anchor. Most GPI-anchored proteins detach from the membrane through cleavage of the glucosamine-mannose bond in the GPI remnant and cross-link with β-1,6-glucan, and then with β-1,3-glucan or chitin [9]. ECM33, a GPI-anchored protein from the SPS2 family in *Saccharomyces cerevisiae*, plays a key role in maintaining cell wall integrity and is conserved across fungi such as *Candida albicans* and *Aspergillus fumigatus* [10, 11]. In *C. albicans*, *ecm33* mutants display increased cell roundness and size, hypersensitivity to cell wall stressors like Congo red and Calcofluor white, and impaired conidial separation and adhesion [10]. In *A. fumigatus*, mutant conidia show increased resistance to phagocytosis, while mycelia are more vulnerable to neutrophil attacks [11]. ECM33 also influences protein glycosylation and secretion. In *C. albicans*, *ecm33* deletion mutants alter the secretome, impacting levels of virulence factors such as SAP2 and disrupting protein export [12]. Furthermore, ECM33 is linked to the TOR signaling pathway, with its deletion enhancing rapamycin sensitivity and activating starvation-induced responses that impair cell proliferation [12]. Overall, ECM33 is essential not only for maintaining fungal cell wall structure and stress resistance but also for regulating critical processes such as secretion, glycosylation, and growth signaling.

Over the past few decades, RNA interference (RNAi) technology has been employed to explore the biology of various biotrophic phytopathogenic fungi, including powdery mildews and rusts [13]. RNAi is an evolutionarily conserved process found in eukaryotic organisms, where it serves as a key regulator in silencing gene expression [14]. Recent studies have demonstrated that fungal pathogens such as *Fusarium graminearum*, *Botrytis cinerea*, and *Sclerotinia sclerotiorum* can efficiently uptake environmental double-stranded RNAs (dsRNAs), which are processed into small interfering RNAs (siRNAs) that silence pathogen genes with complementary sequences [15–17]. These findings have led the development of a crop protection strategy known as spray-induced gene silencing (SIGS), which involves the exogenous application of dsRNAs to plants to silence essential pathogen genes. The effectiveness of SIGS has been studied across various fungal pathogens [15, 16]. Recently, our research group published reports highlighting SIGS’ potential against cucurbit powdery mildew [18, 19].

In this work, we delve into the monosaccharide composition of the *P. xanthii* cell wall and uncover the molecular mechanisms by which ECM33 contributes to cell wall stability and integrity in this phytopathogenic fungus. For the first time, we demonstrate the specific binding capacity of PxECM33 to β-glucans, chitin, and mannans through its heterologous expression and carbohydrate binding assays, highlighting its pivotal role in fungal cell wall assembly. In addition, RNAi assays and microscopy analyses reveal the crucial function of PxECM33 in organizing and maintaining the fungal cell wall, acting as an essential structural element that links the outer and inner layers of the wall, thereby reducing the exposure of cell wall components to degradation by plant enzymes. This underscores the importance of ECM33 for the proper growth and virulence of the fungus. Our findings not only provide valuable insights into fungal biology but also pave the way for innovative antifungal strategies targeting cell wall components. Notably, SIGS directed at the *PxEcm33* gene significantly impacts fungal growth and disease development. This provides a novel tool for developing RNAi-based strategies to control powdery mildew in cucurbits and potentially other economically important crops.

## Results

### Chitin, mannans, and glucans are the main glycosidic components of the *P. xanthii* cell wall

To characterize the cell wall of *P. xanthii*, we analyzed its sugar composition using High-Performance Anion-Exchange Chromatography with Pulsed Amperometric Detection (HPAEC-PAD). The analysis revealed a substantial presence of glucosamine (GlcN), mannose (Man), and glucose (Glc), with GlcN being the most abundant, accounting for 40.9% the total sugar contents. This was followed by Man (30.5%) and Glc (26.08%). Galactose was detected in trace amounts (<2%), while other sugars—fucose, rhamnose, arabinose, xylose, galacturonic acid, and glucuronic acid—were not detected (Table 1). The cell wall also exhibited a high total protein content, reaching 52% of its dry weight. To investigate whether this composition reflects the fungus’s metabolic potential, we analyzed the genome of *P. xanthii* isolate 2086, a hybrid Illumina–PacBio assembly yielding a 142-Mb genome in 1727 scaffolds (N50 = 163 kb), with 14 911 predicted genes and 76.2% repetitive content [20], similar to other powdery mildew genomes. This analysis revealed 157 genes encoding Carbohydrate-Active enZymes (CAZymes), distributed across 76 functional modules. Manual curation indicated that 124 of these genes are likely involved in cell wall metabolism (Table 1, Table S1). The majority were linked to GlcN (27 genes), Man (46 genes), and Glc (35 genes), aligning with the biochemical findings. Interestingly, although galactose, rhamnose, and xylose were detected only in minimal amounts or not detected, the presence of their corresponding metabolic genes (7, 6, and 3 genes, respectively), suggests that these sugars may still play functional roles, potentially contributing to fungal development or pathogenicity.

**Table 1:** Chemical composition of *P. xanthii* cell wall monosaccharides and associated biosynthetic genes.

**Monosaccharides**	**Chemical analysis**		***In silico* analysis**	
	**Amount** ^**a**^	**Relative proportion (%)**	**Number of biosynthetic genes**	**Relative proportion (%)**
Glucosamine	67.62 ± 12.43	40.89	27	21.77
Mannose	50.59 ± 13.08	30.47	46	37.1
Glucose	43.47 ± 7.73	26.08	35	28.23
Galactose	3.26 ± 0.31	1.72	7	5.65
Fucose	ND^b^		ND	
Rhamnose	ND		6	4.84
Xylose	ND		3	2.42
Uronic acids	ND		ND	
Arabinose	ND		ND	

a Data are micrograms of monosaccharide per milligram of cell wall dry weight ± SD from three independent measurements.

b Not detected.

### Sequence analysis, protein modeling, and pocket prediction reveal a potential polysaccharide-binding site in *P. xanthii* ECM33

**Figure 1:**
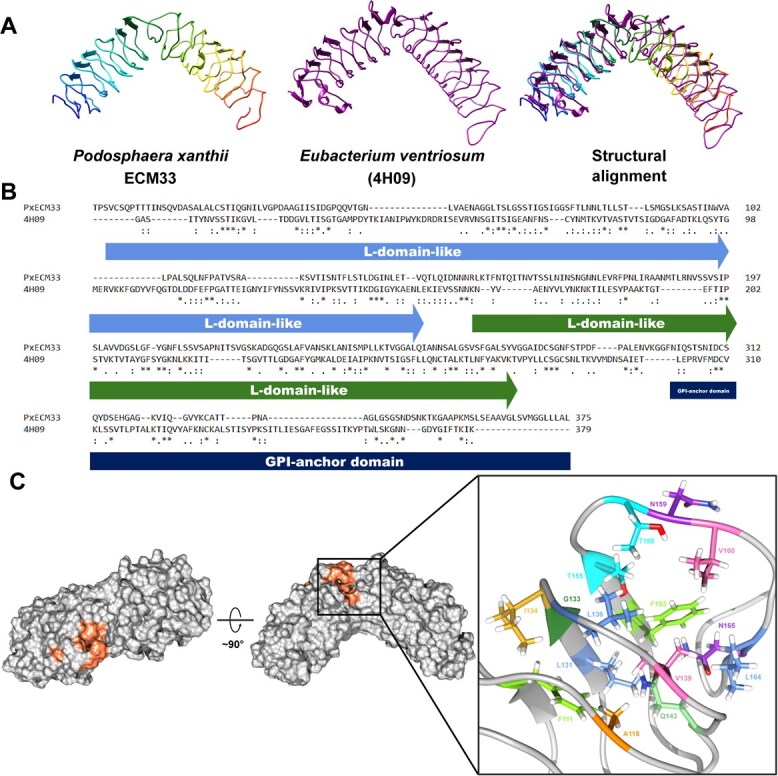
Predicted structural features of the PxECM33 protein. (A) 3D models of *P. xanthii* ECM33 predicted using the I-TASSER server, shown alongside the most structurally similar protein—a hypothetical leucine-rich repeat protein from *E. ventriosum* (PDB 4H09). The structural alignment of both models is also shown. (B) Amino acid sequence alignment between PxECM33 and the EUBVEN_01088 leucine-rich repeat protein from *E. ventriosum*. Conserved residues are marked with asterisks, and L-domain–like regions are indicated with arrows. Alignment was performed using ClustalW via the UniProt server. (C) P2Rank pocket prediction indicating the top-ranked ligand-binding site on the I-TASSER model of PxECM33. The predicted binding site includes the following amino acid residues : F111, A118, L131, G133, I134, L136, V139, Q143, F153, T155, T158, N159, V160, L164, and N165.

In addition to polysaccharides, proteins are essential components of the fungal cell wall. Among these, the GPI-anchored protein ECM33 plays a critical role in maintaining cell wall integrity and is vital for fungal development in both yeast and filamentous fungi [11]. To identify the *P. xanthii* ortholog of ECM33, we examined the available transcriptomic and genomic resources for *P. xanthii* [20, 21]. Building on this data, we identified the *P. xanthii* ortholog, named *PxECM33* (PX138718.1), in the contig_1198 of the genome assembly previously described [20], which encodes a 393-amino acid protein with an estimated molecular weight of ~40 kDa. Despite its importance, ECM33 is a poorly characterized protein, lacking both a crystal structure and annotated functional domains. To infer its function, we constructed 3D structural models using the I-TASSER (Fig. 1A) and AlphaFold2 (Fig. S1A) servers. Both tools generated highly similar structural predictions (Fig. S1A). These models revealed strong similarity to a leucine-rich repeat protein (EUBVEN_01088) from *Eubacterium ventriosum* (PDB: 4H09), despite a low sequence identity of 22.7% (Fig. 1B). Sequence analysis revealed a 54-bp region encoding a signal peptide at the N-terminus (cleavage site at residues 17–18) and a GPI-anchor signal at the C-terminus, with the predicted omega site located at amino acid 369 (Fig. 1B). Further analysis using InterPro identified two L-domain-like regions spanning amino acids 4–139 and 149–276 (Fig. 1B), which are typically involved in protein–protein interactions and ligand recognition [22]. Additionally, I-TASSER predicted potential ligand-binding sites for fungal cell wall components such as mannose, N-acetyl-glucosamine, and D-glucose, suggesting possible interactions with cell wall polysaccharides. To investigate the presence of a ligand-binding pocket, we employed P2Rank. This analysis identified a surface-exposed binding pocket comprising residues Phe111, Ala118, Leu131, Gly133, Ile134, Leu136, Val139, Gln143, Phe153, Thr155, Thr158, Asn159, Val160, Leu164, and Asn165 (Fig. 1C).

### Protein-binding assays reveal polysaccharide-binding properties of the recombinant protein PxECM33

**Figure 2:**
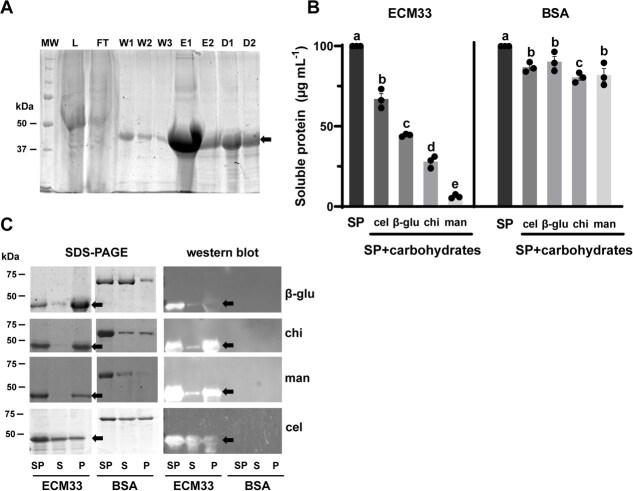
Polysaccharide-binding activity of His-tagged PxECM33. (A) *In vitro* expression and purification of His-tagged PxECM33. SDS-PAGE analysis shows protein samples collected at various purification stages. Arrows indicate bands corresponding to the purified His-tagged PxECM33. Lane labels: L, supernatant of the cell lysate; FT, flowthrough (unbound fraction); W1–W3, first to third wash steps; E1–E2, first and second elution fractions; D1–D2, desalted protein from E1 and E2, respectively. MW, molecular weight marker (Precision Plus Protein™ Standards, Bio-Rad). Arrow indicates PxECM33 band. (B) Polysaccharide-binding assay of His-tagged PxECM33 (100 μg ml^−1^). Soluble PxECM33 (SP) was incubated for 2 h with β-glucan (β-glu), chitin (chi), mannan (man), or cellulose (cel) at a final concentration of 100 μg ml^−1^. BSA was used as a negative-binding control. Bars represent mean ± SE of soluble protein concentration remaining in the supernatant after incubation, based on three technical replicates from three independent experiments. (C) SDS-PAGE and western blot analysis of PxECM33 in supernatant (S) and pellet (P) fractions from the binding assay in (B). Lane labels: SP, PxECM33 soluble protein; S, supernatant after incubation with each polysaccharide; P, pellet after incubation. Arrows indicate PxECM33 bands.

ECM33 has previously been implicated in fungal cell wall organization [10, 11]. To explore its potential carbohydrate-binding capacity, as predicted by I-TASSER, recombinant PxECM33 was expressed in *Escherichia coli* and purified via Ni-NTA affinity chromatography (Fig. 2A). A binding activity assay was then conducted using β-glucans, chitin, and mannans, major structural polysaccharides of the *P. xanthii* cell wall as inferred from the monosaccharide compositional analysis, together with cellulose included as an additional carbohydrate. The recombinant protein was incubated with each carbohydrate separately in washing buffer (pH 8) at 4°C for 2 h. Following incubation, the soluble protein fraction was quantified by measuring absorbance at 280 nm. Binding was strongest with mannans, followed by chitin and β-glucan, supporting the proposed structural role of ECM33 in cell wall organization (Fig. 2B). Sodium dodecyl sulfate–polyacrylamide gel electrophoresis (SDS-PAGE) and western blot analyses (Fig. 2C) further confirmed interactions with all three saccharides. Additionally, although PxECM33 was also observed in the pellet in the presence of cellulose, the protein was predominantly recovered in the supernatant (Fig. 2B and C). These results were consistent with the structural predictions generated by I-TASSER.

### Molecular docking identifies two interaction sites between PxECM33 and fungal cell wall polysaccharides

To further evaluate the polysaccharide-binding capacity of PxECM33, molecular docking analyses were conducted using AutoDock Vina 1.1.2, with β-glucan, chitin, and mannan oligosaccharides as ligands (Fig. S2). Blind docking was conducted independently for each oligosaccharide, generating nine binding models per ligand. For β-glucan, three distinct binding sites were identified, with the most favorable model characterized by the lowest predicted binding free energy (ΔG = −6.3 kcal/mol) and four hydrogen bonds, overlapping with a P2Rank-predicted binding site. This pocket involved key residues Phe153, Asn159, and Asn171 (Fig. S2; Table S2). Similarly, docking with chitin revealed four potential binding sites, distributed near regions overlapping those found with β-glucan. The most favorable chitin-binding model (ΔG = −8.9 kcal/mol) was located near the C-terminus and did not coincide with the P2Rank-predicted pocket. This site involved Ala243, Asn244, and Ser267. In contrast, mannan docking revealed two potential binding sites, with the lowest energy model (ΔG = −7.0 kcal/mol) aligned with the P2Rank-predicted pocket. This pocket included Phe153, Thr161, Asn165, and Asn167 and formed up to five hydrogen bonds (Fig. S2; Table S2). Notably, both β-glucan and mannan docking models reinforced the relevance of the P2Rank-predicted pocket, consistently highlighting residues Phe153, Asn159, and Asn165. Given this convergence, targeted docking simulations were performed to evaluate whether this pocket could also accommodate chitin (Fig. S3). These simulations confirmed a thermodynamically stable interaction, albeit with slightly higher binding energy values compared to blind docking. Chitin formed up to three hydrogen bonds with residues Asn159, Asn165, and Ser163 within the predicted pocket (Fig. S3; Table S2). In parallel, targeted docking analyses were also performed on the P2Rank-predicted pocket for β-glucan and mannan, yielding results comparable to those obtained from blind docking (Fig. S3; Table S2).

### RNAi-mediated silencing of *PxECM33* significantly reduces fungal growth

**Figure 3:**
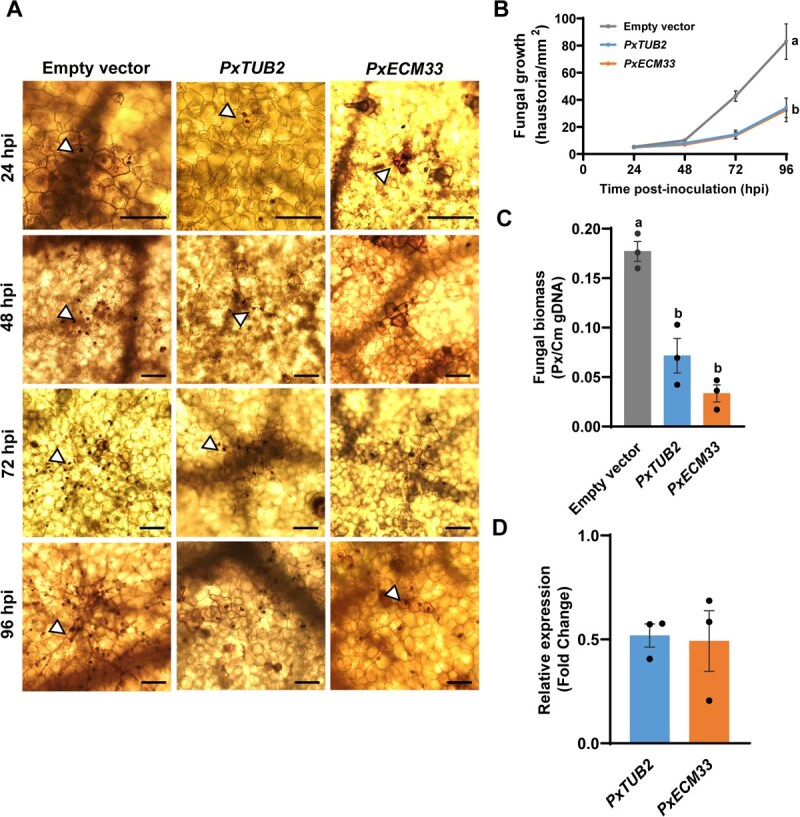
Effect of dsRNA infiltration on *P. xanthii* growth. (A) Melon cotyledons were infiltrated with dsRNA-targeting *PxECM33* and subsequently inoculated with *P. xanthii*, with dsRNA derived from an empty vector and dsRNA targeting *PxTUB2* serving as negative and positive controls, respectively. Fungal structures were visualized using the DAB uptake method. Images were captured at 24, 48, 72, and 96 hpi with *P. xanthii*. Arrowheads indicate haustoria. Scale bars: 100 μm. (B) Quantification of fungal growth by haustorial counting. Growth is expressed as the number of haustoria per mm^2^ of infiltrated tissue. Data represent the mean ± SE of 30 samples from three independent experiments. (C) Molecular estimation of fungal biomass by qPCR. Samples were collected at 96 hpi for genomic DNA extraction. Fungal biomass was assessed by calculating the ratio of *P. xanthii* to melon genomic DNA (Px/Cm gDNA), based on amplification of *PxTUB2* and *CmACT7*. Data represent the average of three technical replicates from three biological replicates ± SE. (D) Assessment of dsRNA-induced gene silencing efficiency by RT-qPCR. Total RNA was extracted from dsRNA-infiltrated melon cotyledons at 96 hpi. Gene expression changes are shown as fold-change relative to the empty vector control, normalized to *PxEF1* and *CmACT7*. Data represent mean values from three independent experiments ± SE. Values sharing the same letter in (B), (C), and (D) are not significantly different (*P* = 0.05, Fisher’s LSD test).

To elucidate the role of PxECM33 in melon–*P. xanthii* interactions, RNAi-based gene silencing assays were conducted using dsRNA molecules following the method described by Ruiz-Jiménez *et al*. [18]. Melon cotyledon discs were treated with different dsRNA concentrations, air-dried, and inoculated with *P. xanthii* conidia (10^5^ spores ml^−1^). As a negative control, nonspecific dsRNAs containing multiple cloning site sequences were synthesized from an empty pL4440 vector. As expected, these dsRNAs had no significant effect on fungal development relative to the water-treated control. A dsRNA targeting the constitutively expressed *PxTUB2* gene (β-tubulin) served as the positive control. While the negative controls had no observable effect, treatment with PxECM33-dsRNA markedly suppressed disease symptoms (Fig. S4). Notably, even the lowest concentration tested (100 ng ml^−1^) produced a strong inhibitory effect, suggesting high silencing efficacy. To validate these findings, similar experiments were performed on melon cotyledons, where infiltration with 100 ng ml^−1^ dsRNA preceded fungal inoculation. Fungal biomass quantification, both by haustorium counts under a light microscope (Fig. 3A and B) and by quantitative PCR (qPCR) (Fig. 3C), revealed significant reductions in pathogen growth. Reverse transcription quantitative PCR (RT-qPCR) analysis further confirmed a ~50% decrease in *PxECM33* transcript levels (Fig. 3D), linking gene silencing directly to diminished fungal proliferation. Collectively, these results demonstrate that RNAi-mediated silencing of *PxECM33* effectively impairs fungal growth and disease progression, positioning it as a promising molecular target for the control of *P. xanthii* infections.

### PxECM33 is essential for maintaining proper cell wall architecture

**Figure 4:**
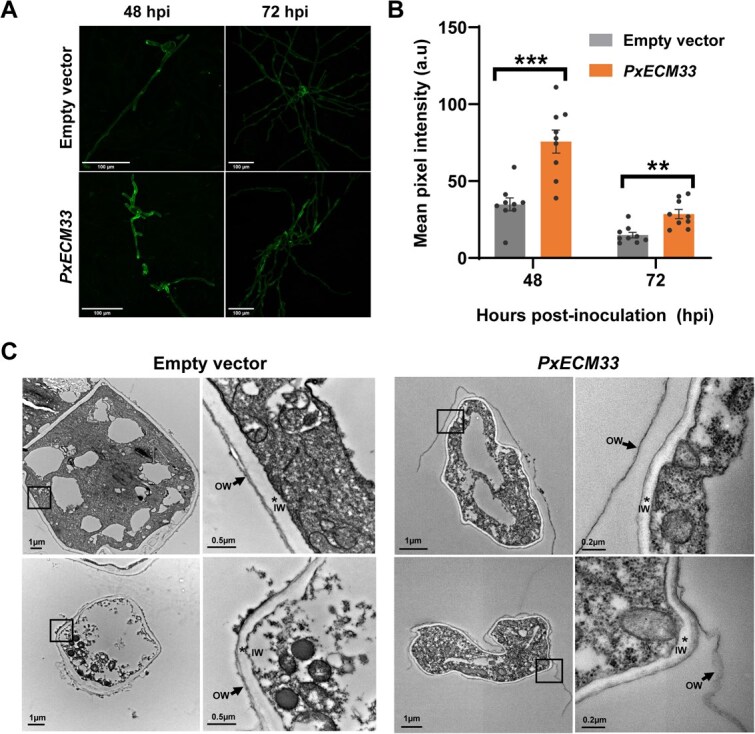
Silencing of *PxECM33* induces morphological abnormalities in the fungal cell wall. (A) Representative confocal laser scanning microscopy images showing chitin-associated fluorescence in *P. xanthii* hyphae treated with either empty vector (negative control) or PxECM33-dsRNA at 48 and 72 hpi. Chitin was visualized using WGA conjugated to a fluorescent dye. (B) Quantification of chitin. Fluorescence intensity from chitin-conjugates was measured using Fiji software. A line was drawn along the hyphal wall in maximum Z-projections, and fluorescence intensity was recorded along this line. The average signal at each point was calculated across replicates and plotted. Error bars represent the SEM; asterisks indicate statistically significant differences (*P* < 0.05, Student’s *t*-test). (C) TEM images of *P. xanthii* hyphae from infected melon cotyledons at 144 hpi. Compared to controls, PxECM33-silenced hyphae exhibit disorganization of the cell wall, with visible separation between the inner wall and outer wall. Left panels show low-magnification views; boxed areas are shown at higher magnification on the right. Arrowheads indicate the outer cell wall (OW) and asterisks the inner cell wall (IW). Scale bars are shown in each image.

Given the morphological defects observed in *ecm33* mutants of *A. fumigatus* and *C. albicans* [10, 11], we examined *P. xanthii* hyphal morphology following *PxECM33* silencing. Fungal cell wall alterations were assessed using wheat germ agglutinin conjugated to Alexa Fluor 488 (wheat germ agglutinin (WGA)-AF488), a fluorescent chitin-specific marker (Fig. 4A). Fluorescence analysis revealed a pronounced increase in the chitin-binding signal in *PxECM33*-silenced hyphae compared with the empty vector control (Fig. 4B), indicating increased chitin deposition and/or exposure and cell wall remodeling, consistent with previous observations [11]. To further investigate these changes, transmission electron microscopy (TEM) was performed on infected melon cotyledons treated with PxECM33-dsRNA. In the control samples, the fungal cell wall exhibited a well-organized, layered architecture: an electron-transparent inner layer, likely composed of β-glucan and chitin, and an electron-dense outer layer primarily made of mannoproteins (Fig. 4C), as supported by chemical analysis. In contrast, silencing *PxECM33* caused pronounced cell wall disorganization, particularly in the mannoprotein-rich outer layer, which appeared detached from the inner cell wall or was missing in several regions (Fig. 4C). In the silenced samples, the inner layer also appeared more clearly defined and whiter, likely due to increased chitin deposition, consistent with the enhanced fluorescence signal observed previously. The structural compromise of the mannoprotein layer likely hinders fungal development and pathogenicity. Additionally, its disruption may expose inner wall components such as β-glucans and chitin to the host, potentially activating plant immune responses. Together, these findings underscore the critical role of *PxECM33* in preserving cell wall integrity and facilitating fungal virulence.

### Silencing of *PxECM33* activates an early immune response in the host plant

**Figure 5:**
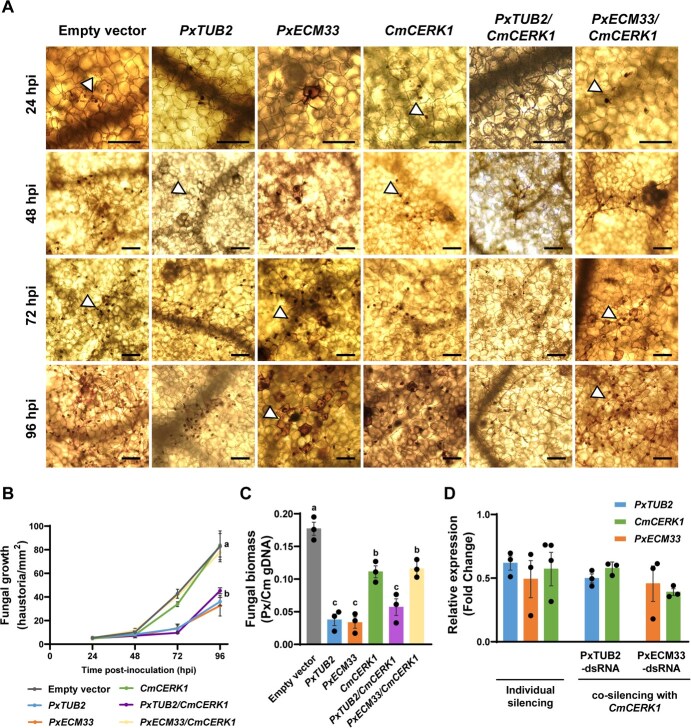
Effect of cosilencing *PxECM33* and the melon immune coreceptor gene *CmCERK1*. (A) Melon cotyledons were infiltrated with dsRNAs targeting *PxECM33*, *CmCERK1*, or both, followed by inoculation with *P. xanthii*, with dsRNA derived from an empty vector and dsRNA targeting *PxTUB2* serving as negative and positive controls, respectively. Cosilencing of *PxTUB2* and *CmCERK1* was also included as an additional control. Fungal structures were visualized using the DAB uptake method. Images were captured at 24, 48, 72, and 96 hpi with *P. xanthii*. Arrowheads indicate haustoria. Scale bars: 100 μm. (B) Quantification of fungal growth by haustorial counting. Growth is expressed as the number of haustoria per square millimeter of infiltrated tissue. Data represent the mean ± SE of 30 samples from three independent experiments. (C) Molecular estimation of fungal biomass by qPCR. Samples were collected at 96 hpi for genomic DNA extraction. Fungal biomass was assessed by calculating the ratio of *P. xanthii* to melon genomic DNA (Px/Cm gDNA), based on amplification of *PxTUB2* and *CmACT7*. Data represent the average of three technical replicates from three biological replicates. (D) Evaluation of dsRNA-induced gene silencing efficiency by RT-qPCR. Total RNA was extracted from dsRNA-infiltrated melon cotyledons at 96 hpi. Gene expression changes are shown as fold-change relative to the empty vector control, normalized to *PxEF1* and *CmACT7*. Bars represent mean values from three biological replicates ± SE. Values sharing the same letter in (B), (C), and (D) are not significantly different (*P* = 0.05, Fisher’s LSD test).

To investigate whether PxECM33 contributes to evading plant immune detection by masking pathogen-associated molecular patterns (PAMPs) such as β-glucan and chitin, we cosilenced *PxECM33* and the melon immune coreceptor gene *CmCERK1* using dsRNA constructs (Fig. 5). CERK1 functions as a coreceptor that perceives multiple carbohydrate elicitors, including chitin and β-glucans [23, 24]. If *PxECM33* silencing activates plant defenses through recognition of cell wall-derived PAMPs, then cosilencing *PxECM33* and *CmCERK1* would be expected to restore fungal growth. As shown in Fig. 5A, the cosilencing treatment restored fungal growth, evaluated by haustorial counting (Fig. 5B), to levels similar to those observed in the empty vector and *CmCERK1* silencing controls, suggesting that *PxECM33* suppression may increase PAMP exposure and thereby trigger early immune activation. Molecular quantification of fungal biomass revealed no significant difference between the *CmCERK1*-silenced group and the cosilenced group (Fig. 5C), further supporting this hypothesis. RT-qPCR confirmed that both *PxECM33* and *CmCERK1* transcript levels were reduced by ~50% in their respective treatments (Fig. 5D), validating the efficiency of gene silencing. Moreover, cosilencing *CmCERK1* did not alter the *PxTUB2-*silencing phenotype, supporting that the plant defense response triggered by *PxECM33* silencing is specifically linked to CERK1-mediated signaling pathway. Together, these results indicate that PxECM33 helps shield fungal PAMPs from host recognition, and that its silencing accelerates CERK1-dependent immune activation.

### SIGS of *PxECM33* offers an effective strategy for controlling powdery mildew

**Figure 6:**
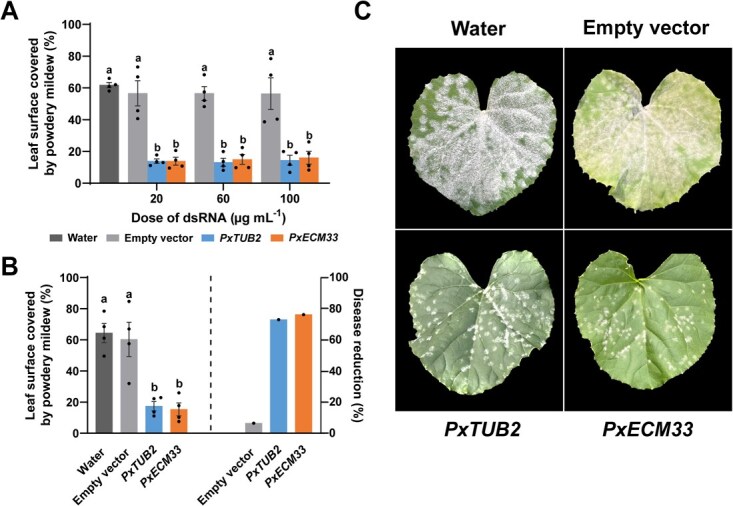
Reduction of powdery mildew symptoms by SIGS. (A) Optimization of dsRNA dosage for SIGS experiments in a growth chamber. The graph shows the effect of SIGS-mediated silencing of *PxECM33* on cucurbit powdery mildew symptom development. Water and dsRNA derived from an empty vector were used as negative controls, while PxTUB2-dsRNA served as a positive control. Melon plants were sprayed with varying doses of dsRNA and inoculated with a fresh suspension of *P. xanthii* conidia (1 × 10^4^ conidia ml^−1^). Disease severity, expressed as the percentage of leaf surface covered by powdery mildew, was assessed 14 days post-inoculation (dpi). Each data point represents the mean of three samples from four independent experiments, with error bars indicating the SEM. Data points sharing the same letter are not significantly different (*P* = 0.05, Fisher’s LSD test). (B) Effect of SIGS on cucurbit powdery mildew symptoms under greenhouse conditions. Melon plants were sprayed with dsRNA (20 μg ml^−1^) and inoculated with a fresh suspension of *P. xanthii* conidia as described above. Disease severity (DS), expressed as the percentage of leaf surface covered by powdery mildew, was recorded 14 dpi. Each data point represents the average of seven biological replicates from four independent experiments, with error bars denoting the SEM. Data points with the same letter are not significantly different (*P* = 0.05, Fisher’s LSD test). The percentage of disease reduction (DR) relative to water control is also shown. (C) Representative images from SIGS experiments. Photographs of sprayed melon leaves were taken at 14 dpi.

To evaluate *PxECM33* as a target for powdery mildew management, a nontransgenic RNA interference approach named SIGS was employed. For this, dsRNAs were applied to the second leaves of melon plants, followed by inoculation with *P. xanthii* conidia 24 h later. Initial experiments were conducted in controlled growth chambers (Fig. 6A), and subsequently under greenhouse conditions (Fig. 6B). In both settings, dsRNA treatments targeting *PxECM33* and the constitutive gene *PxTUB2* (used as positive control) significantly reduced fungal growth, leading to a marked decrease in visible disease symptoms and leaf surface coverage by powdery mildew (Fig. 6C). Across all trials, disease severity was reduced by ~75% compared to water-treated controls. These results demonstrate that SIGS-targeting *PxECM33* is a promising, precise, and environmentally friendly strategy for cucurbit powdery mildew control.

Given its impact on fungal development and virulence, the distribution of *ECM33* orthologues was assessed using BLASTp (Table S3). The analysis revealed strong conservation of *ECM33* across ascomycete fungi, particularly among phytopathogenic species, including powdery mildew fungi. Orthologues were also identified in saprophytic fungi, yeasts, and molds, suggesting a broadly conserved function. Phylogenetic analysis revealed distinct clustering patterns, with a primary group comprising Helotiales species and a secondary group consisting of Erysiphales (Fig. S5). This is notable, as both orders include agriculturally relevant pathogens. Erysiphales encompasses powdery mildew fungi such as *P. xanthii*, while Helotiales includes pathogens like *Monilinia fructicola* (brown rot of stone fruits) and *S. sclerotiorum* (infecting crops such as tomato and potato). The widespread conservation and key role of ECM33 in maintaining fungal cell wall integrity and mediating host–pathogen interactions underscore its value as a strategic target for antifungal interventions, particularly against powdery mildew diseases.

## Discussion

Significant advances in our understanding of fungal cell wall synthesis have facilitated the development of antifungal agents such as echinocandins and nikkomycin Z, which inhibit β-glucan and chitin biosynthesis, respectively, impairing fungal viability [5, 25]. In *P. xanthii*, monosaccharide analysis revealed an enrichment in N-acetylglucosamine, mannose, and glucose, indicating a cell wall composition rich in chitin, mannans, and β-glucans, key polysaccharides essential for structural integrity and host interaction [26]. Notably, the sugar and protein profiles of *P. xanthii* diverge from those of other phytopathogenic fungi such as *F. graminearum* and *B. cinerea* [6], potentially reflecting adaptations to its obligate biotrophic lifestyle. Supporting this, CAZy analysis of the *P. xanthii* genome revealed a broad set of genes involved in monosaccharide synthesis, aligning with biochemical observations. These results mirror findings in other powdery mildew species, where up to 124–135 CAZy genes and 78 functional modules have been identified [27]. The disproportionate allocation of genetic resources toward cell wall biosynthesis likely represents an evolutionary strategy to bolster defense against environmental stress, host immunity, and antifungal agents. Altogether, these insights reaffirm fungal cell wall biosynthesis as a compelling target for antifungal intervention.

*ECM33*, a conserved gene among molds and yeasts, plays a critical role in maintaining cell wall integrity and regulating essential biological processes [10, 28, 29]. It is involved in yeast-to-hypha transition, biofilm formation, and hydrolytic enzyme secretion in *C. albicans* [12]. In *Aspergillus* species, ECM33 contributes to growth, host colonization, and aflatoxin production [30]. In halophilic fungi, it supports osmotic tolerance [31]. Additionally, it modulates MAPK-Pmk1 signaling, glucose uptake, and TORC1 activation [28, 29], underscoring its role in fungal adaptation and pathogenicity. Our findings demonstrate that *PxECM33* encodes a GPI-anchored protein with structural features resembling leucine-rich repeat domains, which are often associated with ligand recognition and molecular interactions. Its 3D model shows high structural similarity to the leucine-rich repeat protein EUBVEN_01088, which is implicated in protein interactions and immune modulation [32]. The observed binding affinity of recombinant PxECM33 to key fungal cell wall polysaccharides—chitin, β-glucans, and mannans—supports its hypothesized involvement in maintaining cell wall architecture. Moreover, *in silico* docking analyses offered molecular-level validation of these interactions, identifying specific ligand-binding sites that suggest a scaffold-like role for PxECM33 in organizing or masking structural wall components. Our results are further supported by recent findings on the DAMP receptor IGP1, which detects cellulose oligomers through a dedicated carbohydrate-binding pocket located within its LRR domain [33, 34]. Work is currently underway in our laboratory to validate these specific ligand-binding sites in PxECM33 using site-directed mutagenesis.

**Figure 7:**
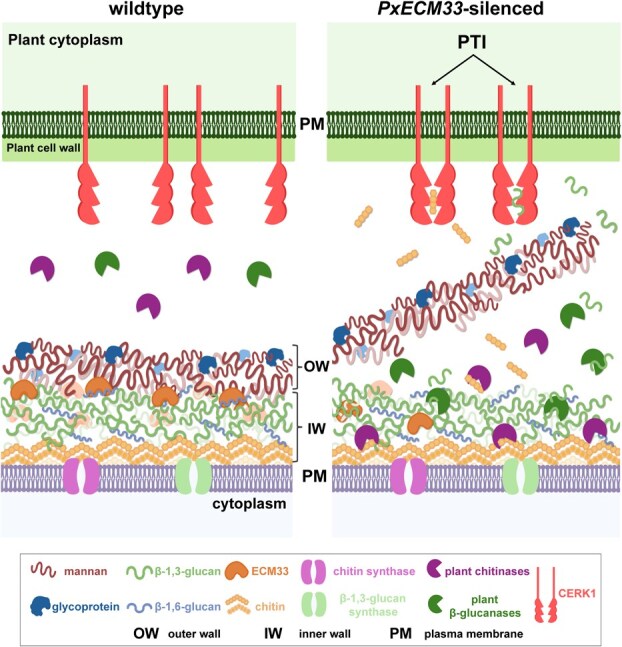
Schematic representation of the proposed role of PxECM33 in the fungal cell wall. (A) When PxECM33 is present, the outer and inner layers of the fungal cell wall remain closely interconnected, restricting plant hydrolases (such as chitinases and β-glucanases) from accessing structural wall components. This limited enzymatic access reduces the release of immunogenic oligomers. (B) In contrast, absence of PxECM33 leads to detachment of the outer wall layer, composed primarily of mannoproteins, from the inner wall. This separation permits plant hydrolases to degrade chitin and β-glucan polysaccharides, resulting in the release of oligomers that are perceived via the CmCERK1 coreceptor, thereby triggering PAMP-triggered immunity (PTI). For clarity, only major components of the fungal cell wall are illustrated.

Silencing of *PxECM33* via RNA interference impaired fungal growth, likely as a consequence of compromised cell wall integrity. The silencing phenotype was characterized by increased chitin-associated fluorescence and a clear separation of the mannoprotein outer layer observed by TEM, consistent with previous reports [11, 35]. Nevertheless, the *PxECM33*-silenced fungus remained infective, as cosilencing *PxECM33* together with the melon immune coreceptor *CmCERK1* restored fungal development. This indicates that the fungus remains viable in the absence of ECM33, in agreement with observations from other systems [11, 35]. CERK1 acts as a coreceptor that perceives multiple carbohydrate elicitors, including chitin and β-glucans [23]. This rescue phenotype therefore suggests that PxECM33 may also contribute to immune evasion by masking immunogenic cell wall components. Disruption of the mannoprotein layer likely exposes β-glucan and chitin, thereby activating pattern-triggered immunity (PTI) in the host and restricting fungal proliferation. These findings highlight a dual role for ECM33 in both structural maintenance and immunomodulation, adding a new dimension to our understanding of host−pathogen interactions (Fig. 7). Collectively, our results support the hypothesis that PxECM33 functions as a structural scaffold within the fungal cell wall matrix. Although further high-resolution immunolocalization studies are needed to define its spatial distribution, orthologous ECM33 proteins have been characterized in entomopathogenic fungi. In *Beauveria bassiana* and *Metarhizium robertsii*, immunogold electron microscopy has detected ECM33 in the cell walls of both hyphae and conidia, consistent with a conserved role in cell wall integrity and modulation of the host−pathogen interface [36].

Our findings underscore the critical role of the *P. xanthii ECM33* gene in fungal development and pathogenicity, positioning it as a promising molecular target for sustainable disease control. As resistance to conventional fungicides continues to rise in powdery mildew species [4], SIGS emerges as an innovative and promising alternative. By reducing chemical inputs and enabling targeted intervention, SIGS offers a sustainable approach to disease management [18]. The successful application of SIGS targeting *PxECM33*, resulting in a 75% reduction in disease symptoms, reinforces its potential. This outcome aligns with previous SIGS research across diverse phytopathogens, including *P. xanthii* [15, 18, 19, 37], and further supports the feasibility of RNA-based, nontransgenic plant protection strategies. Furthermore, the high degree of ECM33 conservation among ascomycetes suggests broad applicability of this approach beyond cucurbit powdery mildew, anticipating similar results, in particular, against other powdery mildew diseases. Importantly, the absence of ECM33 homologs in animals, plants, or bacteria [12] underscores its fungal specificity, reinforcing its suitability as a targeted antifungal strategy.

## Conclusion

This study uncovers a pivotal role for PxECM33 in maintaining fungal cell wall integrity and evading host immune responses, positioning it as a compelling molecular target for next-generation disease management technologies. By combining molecular modeling, functional genomics, and SIGS application, we not only shed light on a previously uncharacterized protein but also offer a practical, sustainable approach to combatting *P. xanthii*. The broad conservation of ECM33 among ascomycetes amplifies the translational potential of our findings, opening avenues for the development of control strategies against the most important powdery mildew diseases.

## Experimental procedures

### Plants, microbes, and growing conditions

Experimental procedures in this study employed isolate 2086 of *P. xanthii* as the representative fungal strain*.* This fungal isolate was propagated on disinfected cotyledons of zucchini (*Cucurbita pepo* L.) cv. Negro Belleza (Semillas Fitó, Spain) and maintained in 8-cm Petri dishes containing Bertrand medium at 24°C under a photoperiod of 16 h light/8 h dark for 1 week [38]. For RNAi gene-silencing assays, melon plants (*Cucumis melo* L.) cv. Rochet (Semillas Fitó), a powdery mildew-susceptible cultivar, were used. These plants were grown in a controlled environment chamber at 24°C with a 16-h light/8-h dark cycle. Plasmid construction and propagation were performed using the *E. coli* strain DH5α, while protein expression was carried out in the BL21-CodonPlus strain. Cultures were grown in lysogeny broth (LB) medium at 37°C, supplemented with ampicillin (100 μg·ml^−1^) or kanamycin (50 μg·ml^−1^) as needed.

### Preparation of cell walls and carbohydrate analysis

To analyze the polysaccharide composition of *P. xanthii* cell walls, mycelium was collected from 2-week-old infected zucchini cotyledons, ground in liquid nitrogen, and lyophilized. Cell wall extraction was performed following the protocol previously described [6]. Two hydrolysis protocols were employed: 6 N HCl (100°C, 17 h) for amino sugar analysis, and 2 N TFA (120°C, 3 h) for neutral sugars and uronic acids. The hydrolysates were filtered, and their monosaccharide profiles were determined by high-performance anion-exchange chromatography with pulsed amperometric detection (HPAEC-PAD) using an LC 930 Compact IC Flex system (Metrohm, Switzerland) fitted with a FlexiPAD detector. All analyses were performed in triplicate. Monosaccharides were quantified using standard curves from commercial standards: fucose, galactose, galactosamine, arabinose, glucose, glucosamine, rhamnose, xylose, mannose, glucuronic acid, and galacturonic acid. Total nitrogen content was determined using the Dumas method with an EA 3000 elemental analyzer (EuroVector, Italy), equipped with a CHN reactor (980°C), GC column (100°C), and thermal conductivity detector. Protein content was calculated by multiplying the nitrogen value by 6.25 [39].

### Identification of putative fungal cell wall genes

The gene set of *P. xanthii* isolate 2086 was derived from a previously published structural annotation [20]. Functional annotation of the proteome was performed using eggNOG-mapper v2 [40]. CAZymes were identified with the dbCAN3 pipeline [39] using HMMER (dbCAN HMMdb and dbCAN-sub HMMdb) and DIAMOND (CAZydb). Only CAZymes identified by at least two of the three methods were retained. These candidates were manually curated using InterProScan 5 [41] to ensure a more accurate selection of genes potentially involved in fungal cell wall metabolism.

### Sequence analyses, protein modeling, molecular docking, and phylogenetic analyses

To identify the signal peptide and delineate the mature form of PxECM33, predictions were made using SignalP 4.1 [42]. The structure of the mature protein was predicted using both I-TASSER [43] and AlphaFold2 [44]. Structural alignments were carried out using Chimera [45]. Amino acid sequence alignments of the mature proteins were generated using Clustal W via the UniProt server [46], and domain predictions were obtained from InterPro [47]. The presence of a ligand-binding pocket was assessed using P2Rank [48]. To identify potential ligand-binding sites in ECM33, molecular docking was performed using AutoDock Vina 1.1.2 [49], applying the ‘accurate’ setting with default parameters. Both blind docking (no predefined binding region) and targeted docking guided by P2Rank pocket predictions were used where appropriate. Orthologous genes in other fungi were identified through BLASTp analysis using the PxECM33 sequence as a query. Phylogenetic analysis was based on reciprocal best-hit BLAST results, multiple sequence alignments using the EMBL-EBI MUSCLE v5 [50], and a maximum likelihood tree constructed in MEGA12 with the LG + G model [51]. Tree robustness was assessed with 100 bootstrap replicates and visualized using iTOL v6 [52].

### Isolation of nucleic acids and cDNA synthesis

Total RNA and genomic DNA were extracted from powdery mildew-infected melon cotyledons. The samples were homogenized in liquid nitrogen using a mortar and pestle. Genomic DNA was isolated using the MasterPure Yeast DNA Purification Kit (Epicentre, US), and total RNA was extracted with TRI Reagent (Sigma-Aldrich), following the manufacturers’ protocols. DNA and RNA concentrations were measured with a NanoDrop 2000 spectrophotometer (Thermo Fisher Scientific, USA). First-strand cDNA synthesis was performed using SuperScript III Reverse Transcriptase (Thermo Fisher Scientific) and the Oligo dT(20) primer (Invitrogen, USA), following the manufacturer’s instructions.

### Plasmid construction

The primers employed for plasmid construction are detailed in Table S4. For *in vitro* expression of the PxECM33 protein, the pET28b(+) vector (Sigma-Aldrich, Germany) was employed. The pL4440 vector was employed to produce dsRNA for RNA interference silencing, as outlined previously [18]. Plasmid propagation was carried out in *E. coli* DH5α, with subsequent confirmation by PCR, restriction digestion, and sequencing (Stab Vida, Portugal). A complete list of plasmids used and constructed in this study is provided in Table S5, with their key features illustrated in Fig. S6.

### Protein expression and purification

For *in vitro* expression of recombinant N-terminally 6 × His-tagged PxECM33 protein, *E. coli* BL21-CodonPlus cells harboring the pPxECM33-EXPRESS expression vector were used. Cultures were grown in LB medium supplemented with kanamycin (50 μg·ml^−1^) at 37°C until reaching an OD_600_ of 0.4, at which point protein expression was induced with 0.5 mM isopropyl β-D-1-thiogalactopyranoside (IPTG) (PamReac AppliChem, Spain). The cultures were then incubated overnight at 18°C on an orbital shaker at 100 rpm. Following the incubation period, the cells were collected via centrifugation at 8000 × g for 10 min at 4°C. Subsequently, the resulting pellet was resuspended in 20 ml of washing buffer composed of 50 mM Tris, 500 mM sodium chloride (pH 8) supplemented with 1 mM phenylmethylsulfonyl fluoride (PMSF), 0.2 mg·ml^−1^ lysozyme, and 1× concentration of cell lytic buffer (Sigma-Aldrich). To facilitate protein extraction from inclusion bodies, the sample was incubated overnight at 60°C in the presence of guanidinium chloride. Lysates were clarified via sonication on ice (3 × 30 s, 80% amplitude), followed by centrifugation at 100 000 × g for 1 h at 4°C. The supernatants were passed through a 0.45-μm membrane filter and subsequently processed via affinity chromatography employing an ÄKTA Start FPLC system (GE Healthcare, USA). Desalting of purified protein fractions was performed using a prepacked Sephadex G-25 M column (GE Healthcare) to exchange the buffer for washing buffer (50 mM Tris, 500 mM sodium chloride, pH 8.0). Protein purification was verified by SDS-PAGE, and concentration was determined using a NanoDrop 2000 spectrophotometer (Thermo Fisher Scientific).

### Binding activity assays

To assess the polysaccharide-binding activity of PxECM33, a sedimentation assay was adapted from a previous work [53]. In brief, 15 μg of recombinant PxECM33 in washing buffer (50 mM Tris, 500 mM sodium chloride, pH 8.0) was incubated with 1.5 mg of β-glucan or mannan (from *S. cerevisiae*, Sigma-Aldrich), chitin (from shrimp shells, Sigma-Aldrich) or cellulose (cotton-linters microcrystalline cellulose, Sigma-Aldrich). The mixtures were then incubated for 2 h at 4°C with continuous shaking at 350 rpm using an orbital shaker. As controls, identical amounts of PxECM33 in buffer alone and bovine serum albumin (BSA) with polysaccharides were included. Following incubation, samples were centrifuged (5 min, 13 000 × *g*), and supernatant protein concentrations were calculated using the Protein Concentration Calculator webserver (https://www.aatbio.com/tools/calculate-protein-concentration), based on A_280_ readings, extinction coefficient, and theoretical molecular weight obtained from the ExPASy server (https://www.expasy.org/). Supernatant and pellet fractions were analyzed by SDS-PAGE, and PxECM33 presence was assessed via western blotting. Proteins were transferred to a PVDF membrane, blocked with 5% milk in TBS, and incubated with Anti-His Tag antibody (1:5000 dilution). Detection was performed using a secondary antibody and Clarity Western ECL Substrate (Bio-Rad, US).

### *In vitro* dsRNA synthesis

dsRNA was synthesized using linearized RNAi silencing vectors as templates *for in vitro* transcription with the HiScribe T7 Quick High Yield RNA Synthesis Kit (New England BioLabs, USA). Quantification of dsRNA was performed using a NanoDrop 2000 spectrophotometer (Thermo Fisher Scientific), and integrity was verified by electrophoresis on a 2% agarose gel. Representative dsRNAs used in this study are shown in Fig. S7.

### dsRNA-mediated gene silencing assays

#### Leaf disc assay

To evaluate the effect of exogenous application of dsRNA on *P. xanthii* growth, a leaf disc assay was conducted as previously described [18]. Cotyledon discs of melon (8 mm diameter) from 8-day-old seedlings were incubated for 3 h in an aqueous solution containing dsRNA and 0.02% Silwet L-77 surfactant (Lehle Seeds, USA). To assess dose dependency, dsRNA concentrations ranging from 100 to 1000 ng·ml^−1^ were tested. Following the treatment, the discs were transferred onto Beltran agar medium and inoculated with 10 μl of a freshly prepared *P. xanthii* conidial suspension (1 × 10^5^ conidia·ml^−1^). Subsequently, the samples were incubated in a controlled growth chamber set to 24°C, with a photoperiod of 16 h light/8 h dark for 8 days. Disease severity was quantified by estimating the percentage of disc surface covered by powdery mildew using Fiji software [54]. Water-treated discs served as negative controls, and a nonspecific dsRNA containing multiple cloning site sequences derived from an empty pL4440 vector was included as an additional negative control.

#### Cotyledon infiltration assay

To investigate the effects of *ECM33* silencing on *P. xanthii* development and gene expression, melon cotyledons from 8-day-old plants were infiltrated with dsRNA solutions [18]. Solutions containing 100 ng·ml^−1^ of dsRNA were prepared in infiltration buffer (10 mM MES, 10 mM MgCl₂). The solutions were delivered into the abaxial surface of the cotyledons using a needleless insulin syringe. After 24 h, cotyledons were dusted with freshly prepared *P. xanthii* conidial suspensions (1 × 10^5^ conidia·ml^−1^) and incubated under previously described controlled conditions. As a negative control, cotyledons were infiltrated with dsRNA synthesized from an empty vector.

#### Spray-induced gene silencing assay

To evaluate *PxECM33* as an RNAi target for powdery mildew control, SIGS assays were conducted using 3-week-old melon plants [18]. To determine the optimal dsRNA concentration, PxECM33-dsRNA was diluted in DEPC-treated water to final concentrations of 20, 60, and 100 μg·ml^−1^. Leaves were sprayed to runoff with PxECM33-RNAi dsRNA and incubated for 24 h under the conditions describe above. Afterwards, leaves were inoculated with a conidial suspension of *P. xanthii* (1 × 10^4^ conidia·ml^−1^). Plants were maintained under the same conditions until symptom development, which was subsequently assessed using quantitative image analysis as described above. Negative controls included water or empty vector dsRNA treatments, while dsRNA targeting *PxTUB2* (β-tubulin) served as a positive control. The concentration that yielded the most consistent and substantial reduction in disease symptoms was selected for scaling up in larger greenhouse assays. In these experiments, plants were acclimated under greenhouse conditions for 3 days before treatment.

### RT-qPCR and qPCR

Gene expression levels and molecular quantification of *P. xanthii* biomass were assessed using RT-qPCR and qPCR, respectively. Primer sequences are listed in Table S4 and were designed using Primer3 software [55]. To evaluate the effects of dsRNA on the expression of both *P. xanthii* and melon genes, cDNA was synthesized from RNA extracted from *P. xanthii*-infected melon cotyledons infiltrated with dsRNA at 96 h post-inoculation (hpi). The genes *P. xanthii PxEF1* (translation elongation factor 1α, MK249653) and *C. melo CmACT7* (actin-7, XM_008462689.2) were used as reference genes for normalization [21]. Fungal biomass quantification was conducted using total DNA extracted from infected, dsRNA-infiltrated cotyledons at 96 hpi. The fungal (*PxTUB*2, KC333362) and host (*CmACT7*) gene targets were used to calculate the *P. xanthii/C. melo* genomic DNA ratio following the method previously established [56]. RT-qPCR and qPCR were performed on a CFX384 Touch Real-Time PCR Detection System (Bio-Rad) using SsoFast EvaGreen Supermix (Bio-Rad), with cycling conditions as follows: 95°C for 30 s (enzyme activation), followed by 40 cycles of 95°C for 5 s and 65°C for 5 s. All reactions were performed in triplicate. Amplicon sizes were confirmed by electrophoresis on 2% agarose gels, and data were analyzed using CFX Manager Software (Bio-Rad).

### Visualization of fungal development and haustorial count

Fungal growth and haustoria quantification following gene silencing were assessed using 3,3′-diaminobenzidine (DAB) staining for histochemical detection of hydrogen peroxide (H₂O₂) accumulation, as previously described [57]. Eight-millimeter leaf discs were excised from melon cotyledons that had been previously infiltrated and infected, and samples were collected at 24, 48, 72, and 96 hpi. The discs were incubated overnight at ambient temperature in darkness in a 1 mg ml^−1^ DAB solution (pH 3.8; Sigma-Aldrich). After incubation, discs were cleared in boiling ethanol and examined under an Eclipse E800 light microscope (Nikon, Japan) to visualize hyphae and haustoria, identified by the presence of black spots.

### Confocal laser scanning microscopy and chitin quantification

To examine fungal cell walls, infected leaf discs infiltrated with either empty vector-derived dsRNA or PxEM33-dsRNA were collected at 24, 48, and 72 hpi. Discs were cleared by boiling in 96% ethanol at 95°C, rinsed in PBS for 20 min, and incubated overnight at 4°C in PBS containing 5 μg ml^−1^ WGA–Alexa Fluor® 488 conjugate. Samples were imaged using an LSM880 confocal laser scanning microscope (Zeiss, Germany). Fluorescence excitation was carried out using a 488-nm laser, and fluorescence emission was captured through a 495- to 571-nm bandpass detection window. Chitin signal intensity was quantified in Fiji software by tracing a line along hyphal cell walls, with data collected from nine biological replicates. Images were analyzed using maximum Z-projections, and average fluorescence intensity was calculated per drawn line.

### Transmission electron microscopy

For TEM analysis, samples were initially fixed overnight at 4°C in 2.5% glutaraldehyde prepared in 0.1 M cacodylate buffer (pH 7.2) containing 0.1 M sucrose. Postfixation was performed in 1% osmium tetroxide for 2 h. Dehydration was achieved through a graded acetone series (30%–100%), followed by infiltration in a 1:1 solution of Araldite 502 resin (EMS, UK) and pure acetone for 2 h, and then with 100% resin for additional 2–4 h. Samples were embedded in resin molds and polymerized at 60°C for 48 h. Semithin (1 μm) sections were stained with 0.05% toluidine blue to assess quality and localize *P. xanthii* structures. Ultrathin (70–90 nm) sections were cut with an Om U3 ultramicrotome (Reichert, US), mounted on copper grids, stained with 1% uranyl acetate for 45 min, and observed with a CM-200 transmission electron microscope (Philips, The Netherlands).

### Statistical analysis

When applicable, statistical analyses were conducted using either Fisher’s least significant difference (LSD) test or Student’s *t*-test for independent samples, as appropriate. All analyses were performed using GraphPad Prism 8 software (Dotmatics, USA).

## Supplementary Material

Web_Material_uhag101

## Data Availability

The data supporting this article are available within the article itself and its supplementary material online.

## References

[1] Pérez-García A, Romero D, Fernández-Ortuño D. et al. The powdery mildew fungus *Podosphaera fusca* (synonym *Podosphaera xanthii*), a constant threat to cucurbits. Mol Plant Pathol. 2009;10:153–60 19236565 10.1111/j.1364-3703.2008.00527.x PMC6640438

[2] Pirondi A, Pérez-García A, Battistini G. et al. Seasonal variations in the occurrence of *Golovinomyces orontii* and *Podosphaera xanthii*, causal agents of cucurbit powdery mildew in northern Italy. Ann Appl Biol. 2015;167:298–313

[3] Boissot N, Chovelon V, Rittener-Ruff V. et al. A highly diversified NLR cluster in melon contains homologs that confer powdery mildew and aphid resistance. Hortic Res. 2023;11:uhad256 38269294 10.1093/hr/uhad256 PMC10807702

[4] Vielba-Fernández A, Polonio Á, Ruiz-Jiménez L. et al. Fungicide resistance in powdery mildew fungi. Microorganisms. 2020;8:1431 32957583 10.3390/microorganisms8091431 PMC7564317

[5] Gow NAR, Lenardon MD. Architecture of the dynamic fungal cell wall. Nat Rev Microbiol. 2023;21:248–59 36266346 10.1038/s41579-022-00796-9

[6] Yugueros SI, Peláez J, Stajich JE. et al. Study of fungal cell wall evolution through its monosaccharide composition: an insight into fungal species interacting with plants. Cell Surf. 2024;11:100127 38873189 10.1016/j.tcsw.2024.100127 PMC11170279

[7] Plaza V, Silva-Moreno E, Castillo L. Breakpoint: cell wall and glycoproteins and their crucial role in the phytopathogenic fungi infection. Curr Protein Pept Sci. 2020;21:227–44 31490745 10.2174/1389203720666190906165111

[8] Tada R, Latgé JP, Aimanianda V. Undressing the fungal cell wall/cell membrane—the antifungal drug targets. Curr Pharm Des. 2013;19:3738–47 23278542 10.2174/1381612811319200012

[9] Pittet M, Conzelmann A. Biosynthesis and function of GPI proteins in the yeast *Saccharomyces cerevisiae*. Biochim Biophys Acta Mol Cell Res. 2007;1771:405–20 10.1016/j.bbalip.2006.05.015 16859984

[10] Martinez-Lopez R, Monteoliva L, Diez-Orejas R. et al. The GPI-anchored protein CaEcm33p is required for cell wall integrity, morphogenesis and virulence in *Candida albicans*. Microbiology. 2004;150:3341–54 15470113 10.1099/mic.0.27320-0

[11] Chabane S, Sarfati J, Ibrahim-Granet O. et al. Glycosylphosphatidylinositol-anchored Ecm33p influences conidial cell wall biosynthesis in *Aspergillus fumigatus*. Appl Environ Microbiol. 2006;72:3259–67 16672465 10.1128/AEM.72.5.3259-3267.2006 PMC1472355

[12] Gil-Bona A, Monteoliva L, Gil C. Global proteomic profiling of the secretome of *Candida albicans* ecm33 cell wall mutant reveals the involvement of Ecm33 in Sap2 secretion. J Proteome Res. 2015;14:4270–81 26290404 10.1021/acs.jproteome.5b00411

[13] Padilla-Roji I, Ruiz-Jiménez L, Bakhat N. et al. RNAi technology: a new path for the research and management of obligate biotrophic phytopathogenic fungi. Int J Mol Sci. 2023;24:9082 37240427 10.3390/ijms24109082 PMC10219382

[14] Shabalina SA, Koonin EV. Origins and evolution of eukaryotic RNA interference. Trends Ecol Evol. 2008;23:578–87 18715673 10.1016/j.tree.2008.06.005 PMC2695246

[15] Koch A, Biedenkopf D, Furch A. et al. An RNAi-based control of *Fusarium graminearum* infections through spraying of long dsRNAs involves a plant passage and is controlled by the fungal silencing machinery. PLoS Pathog. 2016;12:e1005901 27737019 10.1371/journal.ppat.1005901 PMC5063301

[16] McLoughlin AG, Wytinck N, Walker PL. et al. Identification and application of exogenous dsRNA confers plant protection against *Sclerotinia sclerotiorum* and *Botrytis cinerea*. Sci Rep. 2018;8:7320 29743510 10.1038/s41598-018-25434-4 PMC5943259

[17] Nerva L, Sandrini M, Gambino G. et al. Double-stranded RNAs (dsRNAs) as a sustainable tool against gray mold (*Botrytis cinerea*) in grapevine: effectiveness of different application methods in an open-air environment. Biomolecules. 2020;10:200 32013165 10.3390/biom10020200 PMC7072719

[18] Ruiz-Jiménez L, Polonio Á, Vielba-Fernández A. et al. Gene mining for conserved, non-annotated proteins of *Podosphaera xanthii* identifies novel target candidates for controlling powdery mildews by spray-induced gene silencing. J Fungi. 2021;7:735 10.3390/jof7090735 PMC8465782 34575773

[19] Bakhat N, Jiménez-Sánchez A, Ruiz-Jiménez L. et al. Fungal effector genes involved in the suppression of chitin signaling as novel targets for the control of powdery mildew disease via a nontransgenic RNA interference approach. Pest Manag Sci. 2025;81:3452–63 39797552 10.1002/ps.8660

[20] Polonio Á, Díaz-Martínez L, Fernández-Ortuño D. et al. A hybrid genome assembly resource for *Podosphaera xanthii*, the main causal agent of powdery mildew disease in cucurbits. Mol Plant-Microbe Interact. 2021;34:319–24 33141618 10.1094/MPMI-08-20-0237-A

[21] Polonio Á, Seoane P, Claros MG. et al. The haustorial transcriptome of the cucurbit pathogen *Podosphaera xanthii* reveals new insights into the biotrophy and pathogenesis of powdery mildew fungi. BMC Genomics. 2019;20:543 31272366 10.1186/s12864-019-5938-0 PMC6611051

[22] Li D, Wu M. Pattern recognition receptors in health and diseases. Signal Transduct Target Ther. 2021;6:1–24 34344870 10.1038/s41392-021-00687-0 PMC8333067

[23] Yang C, Wang E, Liu J. CERK1, more than a co-receptor in plant-microbe interactions. New Phytol. 2022;234:1606–13 35297054 10.1111/nph.18074

[24] Ai Y, Li Q, Li C. et al. Tomato LysM receptor kinase 4 mediates chitin-elicited fungal resistance in both leaves and fruit. Hortic Res. 2023;10:uhad082 37323235 10.1093/hr/uhad082 PMC10266952

[25] Wu Y, Zhang M, Yang Y. et al. Structures and mechanism of chitin synthase and its inhibition by antifungal drug nikkomycin Z. Cell Discov. 2022;8:129 36473834 10.1038/s41421-022-00495-y PMC9726829

[26] Garcia-Rubio R, de Oliveira HC, Rivera J. et al. The fungal cell wall: *Candida*, *Cryptococcus*, and *Aspergillus* species. Front Microbiol. 2020;10:2993 31993032 10.3389/fmicb.2019.02993 PMC6962315

[27] Wu Y, Ma X, Pan Z. et al. Comparative genome analyses reveal sequence features reflecting distinct modes of host adaptation between dicot and monocot powdery mildew. BMC Genomics. 2018;19:705 30253736 10.1186/s12864-018-5069-z PMC6156980

[28] Takada H, Nishida A, Domae M. et al. The cell surface protein gene *ecm33+* is a target of the two transcription factors Atf1 and Mbx1 and negatively regulates Pmk1 MAPK cell integrity signaling in fission yeast. Mol Biol Cell. 2010;21:674–85 20032302 10.1091/mbc.E09-09-0810 PMC2820430

[29] Umekawa M, Ujihara M, Nakai D. et al. Ecm33 is a novel factor involved in efficient glucose uptake for nutrition-responsive TORC1 signaling in yeast. FEBS Lett. 2017;591:3721–9 29029364 10.1002/1873-3468.12882

[30] Chang PK, Zhang Q, Scharfenstein L. et al. *Aspergillus flavus* GPI-anchored protein-encoding *ecm33* has a role in growth, development, aflatoxin biosynthesis, and maize infection. Appl Microbiol Biotechnol. 2018;102:5209–20 29696338 10.1007/s00253-018-9012-7

[31] Pérez-Llano Y, Rodríguez-Pupo EC, Druzhinina IS. et al. Stress reshapes the physiological response of halophile fungi to salinity. Cells. 2020;9:525 32106416 10.3390/cells9030525 PMC7140475

[32] Takkouche A, Qiu X, Sedova M. et al. Unusual structural and functional features of TpLRR/BspA-like LRR proteins. J Struct Biol. 2023;215:108011 37562586 10.1016/j.jsb.2023.108011 PMC12244429

[33] Jiménez-Sandoval P, Broyart C, Kentish O. et al. Glycan recognition by a plant sentinel immune receptor. bioRxiv. 2025;2025.09.28.679030 10.1126/science.aec6740 42721272

[34] Sun G, Wei P, Dai J. et al. IGP receptors act as redox sensors to mediate broad-spectrum disease resistance induced by cellooligomers. bioRxiv. 2025;2025.10.05.680433

[35] Ramos-Viana V, Moller-Hansen I, Kempen P. et al. Modulation of the cell wall protein Ecm33p in yeast *Saccharomyces cerevisiae* improves the production of small metabolites. FEMS Yeast Res. 2022;22:1–11 10.1093/femsyr/foac037 PMC9440718 35922083

[36] Chen Y, Zhu J, Ying SH. et al. The GPI-anchored protein Ecm33 is vital for conidiation, cell wall integrity, and multi-stress tolerance of two filamentous entomopathogens but not for virulence. Appl Microbiol Biotechnol. 2014;98:5517–29 24549768 10.1007/s00253-014-5577-y

[37] Bocos-Asenjo IT, Amin H, Mosquera S. et al. Spray-induced gene silencing (SIGS) as a tool for the management of pine pitch canker forest disease. Plant Dis. 2024;109:49–62 10.1094/PDIS-02-24-0286-RE 39148367

[38] Álvarez B, Torés JA. Cultivo *in vitro* de *Sphaerotheca fuliginea* (Schlecht. ex Fr.), efecto de diferentes fuentes de carbono sobre su desarrollo. Bol Sanid Veg Plagas. 1997;23:283–8

[39] Zheng J, Ge Q, Yan Y. et al. dbCAN3: automated carbohydrate-active enzyme and substrate annotation. Nucleic Acids Res. 2023;51:W115–21 37125649 10.1093/nar/gkad328 PMC10320055

[40] Cantalapiedra CP, Hernández-Plaza A, Letunic I. et al. eggNOG-mapper v2: functional annotation, orthology assignments, and domain prediction at the metagenomic scale. Mol Biol Evol. 2021;38:5825–9 34597405 10.1093/molbev/msab293 PMC8662613

[41] Jones P, Binns D, Chang HY. et al. InterProScan 5: genome-scale protein function classification. Bioinformatics. 2014;30:1236–40 24451626 10.1093/bioinformatics/btu031 PMC3998142

[42] Petersen TN, Brunak S, von Heijne G. et al. SignalP 4.0: discriminating signal peptides from transmembrane regions. Nat Methods. 2011;8:785–6 21959131 10.1038/nmeth.1701

[43] Yang J, Zhang Y. I-TASSER server: new development for protein structure and function predictions. Nucleic Acids Res. 2015;43:W174–81 25883148 10.1093/nar/gkv342 PMC4489253

[44] Jumper J, Evans R, Pritzel A. et al. Highly accurate protein structure prediction with AlphaFold. Nature. 2021;596:583–9 34265844 10.1038/s41586-021-03819-2 PMC8371605

[45] Pettersen EF, Goddard TD, Huang CC. et al. UCSF Chimera—a visualization system for exploratory research and analysis. J Comput Chem. 2004;25:1605–12 15264254 10.1002/jcc.20084

[46] Apweiler R, Bairoch A, Wu CH. et al. UniProt: the universal protein knowledgebase. Nucleic Acids Res. 2004;32:115D–19 14681372 10.1093/nar/gkh131 PMC308865

[47] Paysan-Lafosse T, Blum M, Chuguransky S. et al. InterPro in 2022. Nucleic Acids Res. 2022;51:D418–27 10.1093/nar/gkac993 PMC9825450 36350672

[48] Jendele L, Krivak R, Skoda P. et al. PrankWeb: a web server for ligand binding site prediction and visualization. Nucleic Acids Res. 2019;47:W345–9 31114880 10.1093/nar/gkz424 PMC6602436

[49] Trott O, Olson AJ. AutoDock Vina: improving the speed and accuracy of docking with a new scoring function, efficient optimization and multithreading. J Comput Chem. 2010;31:455–61 19499576 10.1002/jcc.21334 PMC3041641

[50] Madeira F, Madhusoodanan N, Lee J. et al. The EMBL-EBI job dispatcher sequence analysis tools framework in 2024. Nucleic Acids Res. 2024;52:W521–5 38597606 10.1093/nar/gkae241 PMC11223882

[51] Kumar S, Stecher G, Suleski M. et al. Molecular evolutionary genetics analysis version 12 for adaptative and green computing. Mol Biol Evol. 2024;41:1–9 10.1093/molbev/msae263 PMC11683415 39708372

[52] Letunic I, Bork P. Interactive tree of life (iTOL) v6: recent updates to the phylogenetic tree display and annotation tool. Nucleic Acids Res. 2024;52:W78–82 38613393 10.1093/nar/gkae268 PMC11223838

[53] Volk H, Marton K, Flajsman M. et al. Chitin-binding protein of *Verticillium nonalfalfae* disguises fungus from plant chitinases and suppresses chitin-triggered host immunity. Mol Plant-Microbe Interact. 2019;32:1378–90 31063047 10.1094/MPMI-03-19-0079-R

[54] Rueden CT, Schindelin J, Hiner MC. et al. ImageJ2: ImageJ for the next generation of scientific image data. BMC Bioinformatics. 2017;18:529 29187165 10.1186/s12859-017-1934-z PMC5708080

[55] Thornton B, Basu C. Real-time PCR (qPCR) primer design using free online software. Biochem Mol Biol Educ. 2011;39:145–54 21445907 10.1002/bmb.20461

[56] Vela-Corcía D, Bellón-Gómez D, López-Ruiz F. et al. The *Podosphaera fusca TUB2* gene, a molecular “swiss army knife” with multiple applications in powdery mildew research. Fungal Biol. 2014;118:228–41 24528644 10.1016/j.funbio.2013.12.001

[57] Martínez-Cruz J, Romero D, de la Torre FN. et al. The functional characterization of *Podosphaera xanthii* candidate effector genes reveals novel target functions for fungal pathogenicity. Mol Plant-Microbe Interact. 2018;31:914–31 29513627 10.1094/MPMI-12-17-0318-R

