# Supplementary material for: Cracking the wall: the fungal cell wall assembly protein ECM33 is a promising molecular target in powdery mildew fungi
Source: Hortic Res. 2026 Mar 13;13(7):uhag101. doi: 10.1093/hr/uhag101 (PMC13282541; doi:10.1093/hr/uhag101)
Supplement: Web_Material_uhag101 [file web_material_uhag101.zip › Supplemental Tables & Figures Padilla-Roji et al. R1.pdf]

Cracking the wall: The fungal cell wall assembly protein ECM33 is a promising molecular target in powdery mildew fungi

Isabel Padilla-Roji[1,2], Alejandro Jiménez-Sánchez[1,2], Sara Yugueros[3,4], Hugo Mélida[3,4], Álvaro Polonio[1,2], Dolores Fernández Ortuño[1,2], Alejandro Pérez-García[1,2]*

[1]Departamento de Microbiología, Facultad de Ciencias, Universidad de Málaga, Málaga, Spain.
[2]Instituto de Hortofruticultura Subtropical y Mediterránea "La Mayora", Universidad de Málaga, Consejo Superior de Investigaciones Científicas (IHSM−UMA−CSIC), Málaga, Spain.
[3]Área de Fisiología Vegetal, Departamento de Ingeniería y Ciencias Agrarias, Universidad de León, León, Spain.
[4]Instituto de Biología Molecular, Genómica y Proteómica (INBIOMIC), Universidad de León, León, Spain

*Corresponding author: Alejandro Pérez-García (aperez@uma.es)

Table S2. Molecular docking analysis of PxECM33[a] with predominant *P. xanthii* cell wall carbohydrates

	Blind docking				Targeted docking[d]			
**Ligand[b]**	**Residue**	**HB[c]**	**HB distance (Å)**	**Estimated ΔG (kcal/mol)**	**Residue**	**HB**	**HB distance (Å)**	**Estimated ΔG (kcal/mol)**
**β-glucan**	F153	1	2.67	-6.3	F153	1	2.26	-6.6
	N159	1	2.10		N159	1	2.14	
	N171	2	2.19		T161	1	2.50	
			2.14					
**chitin**	A243	1	2.10	-8.9	N159	1	2.11	-8.4
	N244	3	1.85		N165	2	2.68	
			2.42				2.37	
			2.25					
	S267	1	1.88		S163	1	2.18	
**mannan**	F153	2	1.86	-7.0	F153	1	2.09	-7.2
			2.06					
	T161	1	2.02		V160	1	2.17	
	N165	1	2.25		L164	1	1.98	
	N167	1	2.39					

[a] The protein model of PxECM33 predicted by I TASSER was used.

[b] For β-glucan and chitin, trimer and tetramer oligomers were used. For mannan, the structure Man(β1→4)Glc(β1→4)Man(β1→4)β-Tal was used.

[c] HB, hydrogen bonds.

[d] Targeted docking was focused on the binding site pocket predicted by P2Rank.

Table S4. Primers used in this study

Primer name	Sequence[a] (5' -3')
Plasmid construction for protein expression	
PxECM33-EcoRI	CGAATTCGACTCCATCTGTGTGCTC (EcoRI)[b]
PxECM33-NotI	GCGGCCGTTACTTGGTTTTGTTT (NotI)
Plasmid construction for RNAi and dsRNA synthesis	
T7-F	GTTGTAAAACGACGGCCAGT
T7-R	CGAGTCAGTGAGCGAGGAA
PxECM33si-F	TAATGGTACCAGTGCAGACCCTTCAAATCG (KpnI)
PxECM33s*i*-R	TAATCCCGGGTCCTTGCCCATCGGCTTTTG (SmaI)
TUB2F-si	TAATCCATGGATGTTGTTCGGCGTGAAGC (NcoI)
TUB2R-si	TAATAGATCTGAGGTCGCCATATGAAGGG (BglII)
CERK1F-si	TAATCCATGGGGAGGGTGCCAATTTGTCTTTCG (NcoI)
CERK1R-si	TAATAGATCTCTCCACAGGAGCAGTTCAC (BglII)
Gene expression analysis	
EF-1-F	TCAAGAACGTGTCCGTGAAG
EF-1-R	CAATCAAGCACTGGAGCGTA
ACT7-F	TCATGGTGGGTATGGGCCAGAAAG
ACT7-R	TACAGTGATAGAACGGCCTGGATG
RT-PxECM33-F	TCGTGCTGCAAACATGACTC
RT-PxECM33-R	GCGCCTCCAACATAAGACAG
RT-PxTUB2-F	TGACCGTCCCTGAATAAC
RT-PxTUB2-R	GAACGTTGTTGGGATCCAT
RT-CmCERK1-F	TGGTCAAGGTGATGGTATAGC
RT-CmCERK1-R	TCGACCTGTGCCTCGTAAATGTTG
Molecular estimation of fungal growth	
TUB2g-F	TTGTAGGAATCACATCCCTTTCTC
TUB2g-R	TTCTTCCGGTTGCATGGGTGGTTC
Acting-F	GGCTGGATTTGCCGGTGATGATGC
Acting-R	GGAAGGAGGAAATCAGTGTGAACC

Table S5. Plasmids used in this study

Plasmid	Characteristics	Reference
pL4440	Bacterial vector used for *in vitro* synthesis of double-stranded RNA (dsRNA); also used as a negative control in gene silencing experiments.	Kindly donated by Andrew Fire, Stanford University
pPxTUB-RNAi	Silencing vector containing a 324 bp fragment of *PxTUB2,* used as a positive control in gene silencing experiments.	Ruiz-Jiménez *et al. (*2021)
pCmCERK1-RNAi	Silencing vector containing a 337 bp fragment of *CmCERK1,* used to disrupt melon pattern-triggered immunity (PTI) elicited by fungal cell wall fragments.	Bakhat *et al.* (2025)
pPxECM33-RNAi	Silencing vector containing a 285 bp fragment of *PxECM33*.	This study
pET28b(+)	Bacterial vector for expression of N-terminally 6xHis-tagged proteins with a thrombin site.	Sigma-Aldrich, Germany
pPxECM33-EXPRESS	Bacterial expression vector containing the ORF of *PxECM33* without SP or GPI domain.	This study

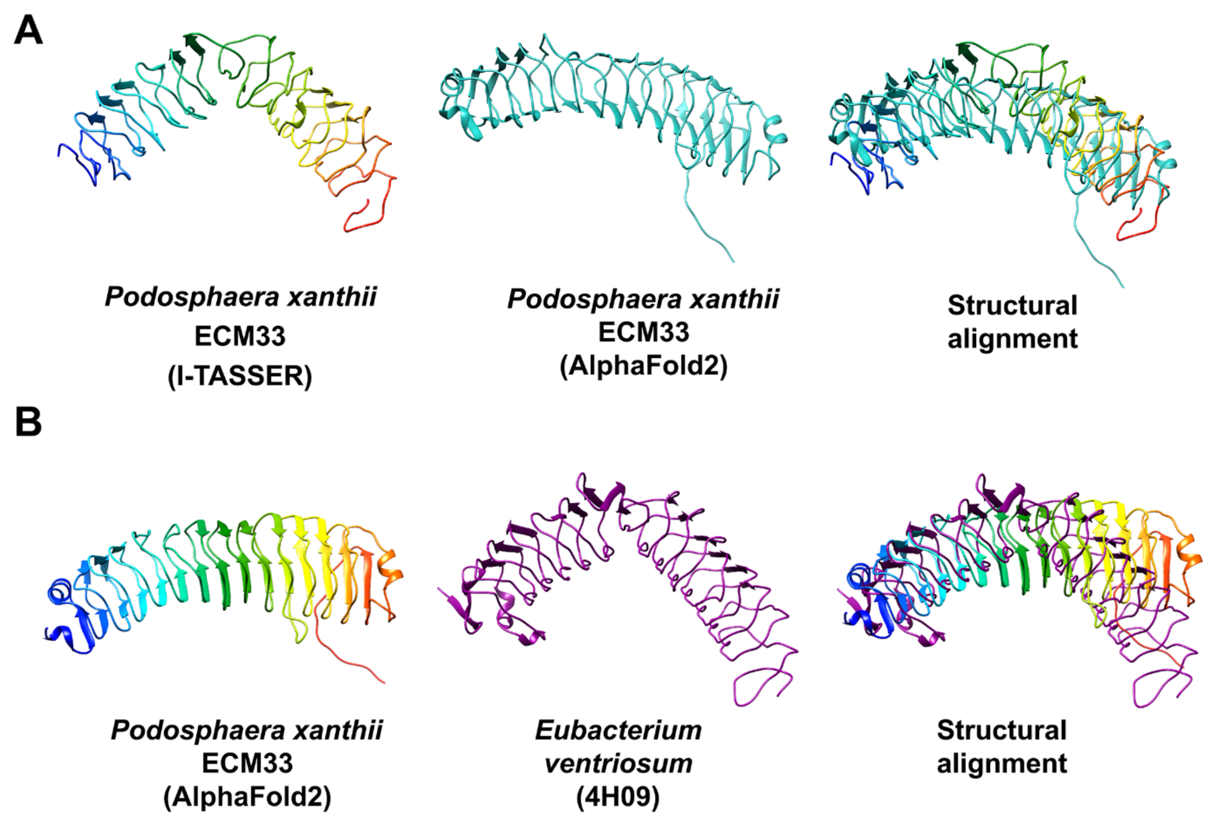

Fig. S1. Structural alignment of PxECM33 with other protein models. (A) Predicted 3D structures of PxECM33 generated using I-TASSER and AlphaFold2. The structural alignment of both models is also shown. (B) Structural alignment of the PxECM33 AlphaFold2 model with its closest structural homolog, a hypothetical leucine-rich repeat protein from *Eubacterium ventriosum* (PDB ID: 4H09).

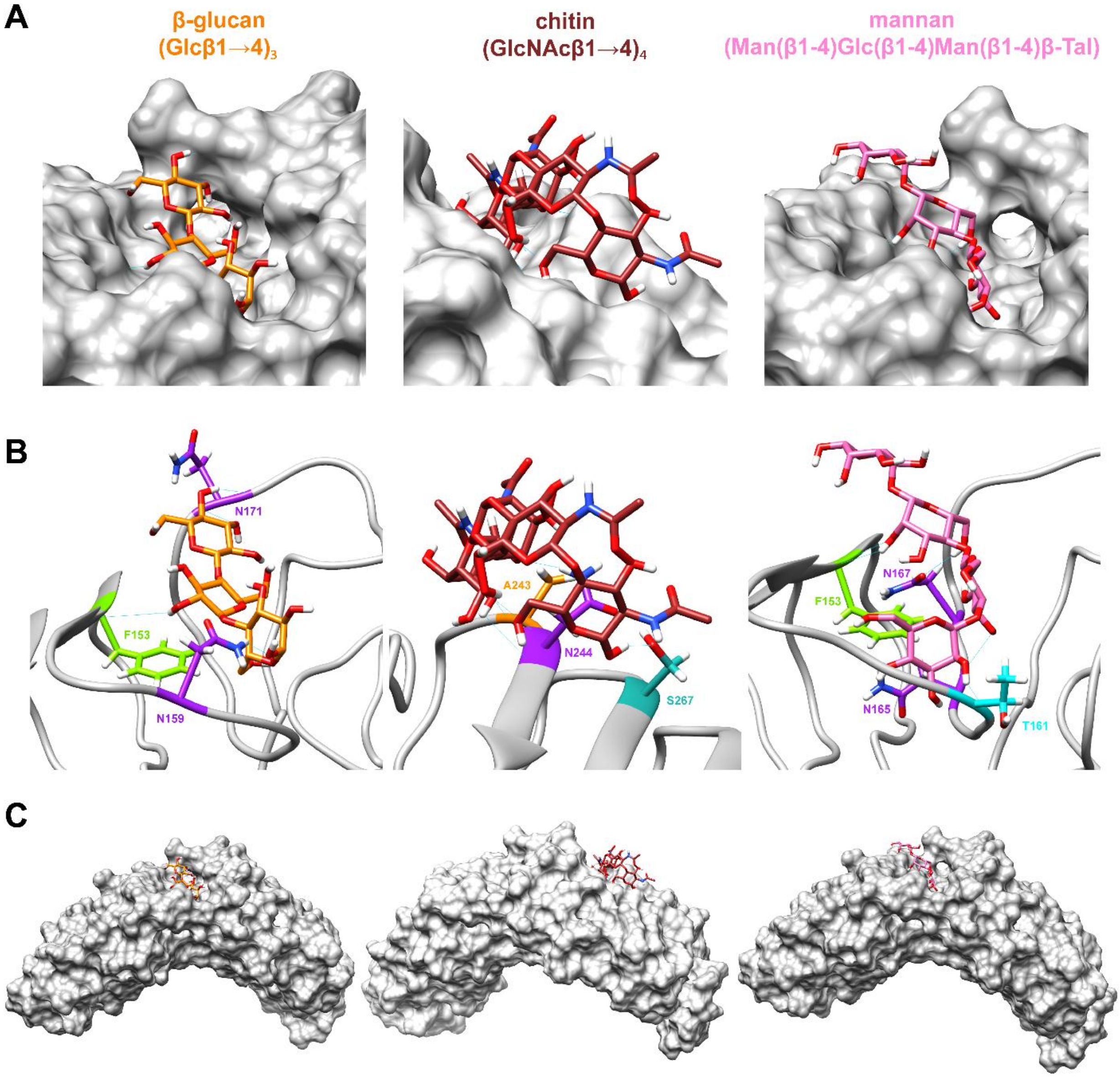

Fig. S2. Molecular blind docking of cell wall carbohydrates to PxECM33. Molecular docking was performed using Chimera 1.18 and the PxECM33 3D model predicted by I-TASSER, with the three most abundant carbohydrates in the *P. xanthii* cell wall: β-glucan, chitin, and mannan. For β-glucan and chitin, trimer and tetramer oligomers were used, respectively. For mannan, the structure Man(β1→4)Glc(β1→4)Man(β1→4)β-Tal was used. (A) Surface representation of PxECM33 highlighting the predicted carbohydrate-binding sites. (B) Predicted binding interactions between PxECM33 and each carbohydrate. β-glucan forms four hydrogen bonds with residues F153 (1: 2.67 Å), N159 (2: 2.10 Å), and N171 (3: 2.19 Å; 4: 2.14 Å). Chitin establishes five hydrogen bonds with A243 (1: 2.10 Å), N244 (2: 1.85 Å; 3: 2.42 Å; 4: 2.25 Å), and S267 (5: 1.88 Å). Mannan forms five hydrogen bonds involving F153 (1: 1.86 Å; 2: 2.06 Å), T161 (3: 2.02 Å), N165 (4: 2.25 Å), and N167 (5: 2.39 Å). Hydrogen bonds are shown as cyan lines, and the interacting residues are labeled in distinct colors. (C) Overview of the docking results showing the spatial arrangement of β-glucan, chitin, and mannan within the proposed binding site of PxECM33.

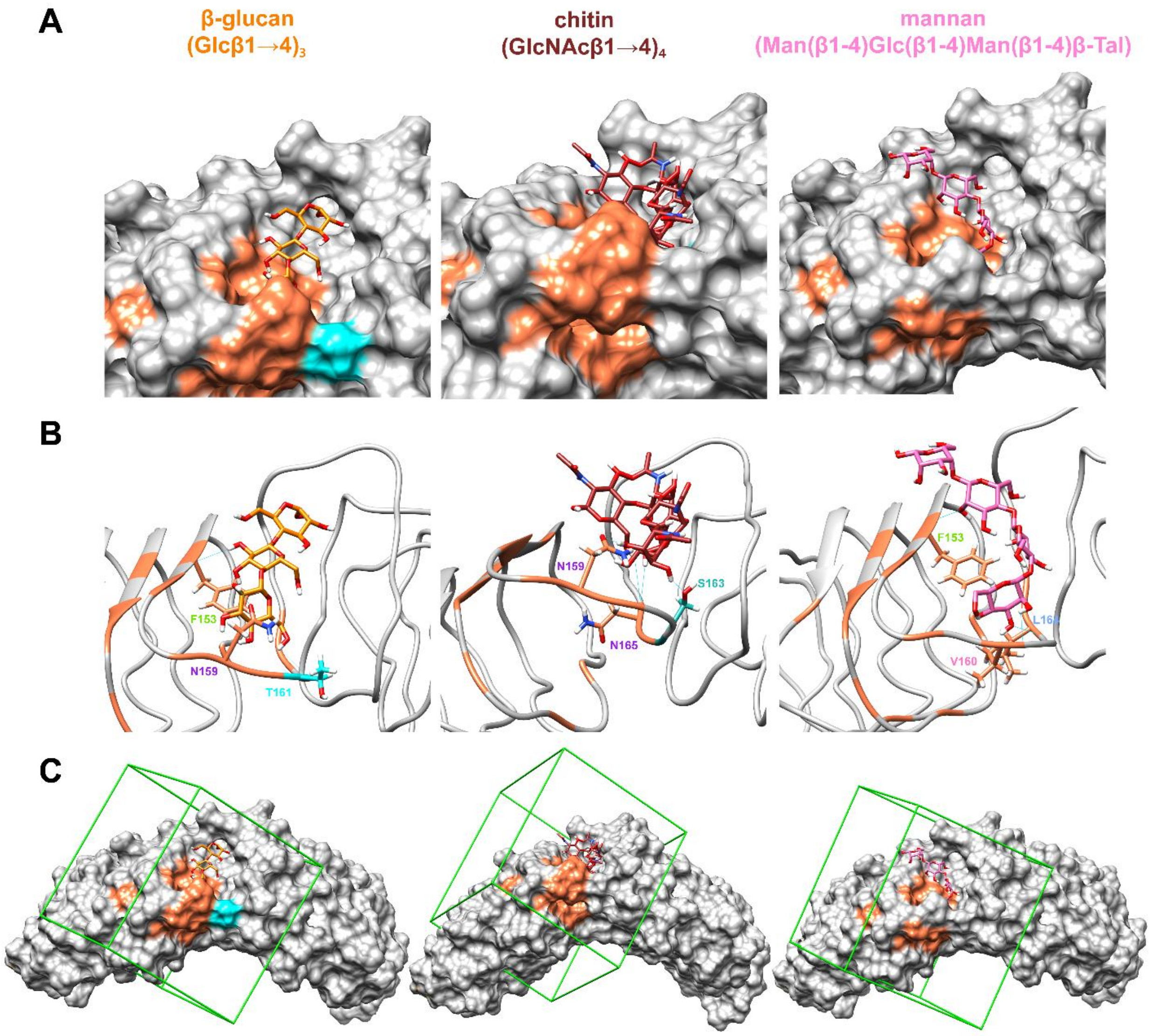

Fig. S3. Molecular targeted docking of cell wall carbohydrates to PxECM33. Molecular targeted docking was performed using Chimera 1.18, focusing on the binding site pocket predicted by P2Rank, with β-glucan, chitin, and mannan. For β-glucan and chitin, trimer and tetramer oligomers were used, respectively. For mannan, the structure Man(β1→4)Glc(β1→4)Man(β1→4)β-Tal was used. (A) Detailed view of the predicted binding pocket for the three analyzed carbohydrates. Amino acid residues identified by P2Rank as part of the binding site are highlighted in orange. (B) Close-up views of the interactions between PxECM33 and individual carbohydrates. β-glucan is predicted to form three hydrogen bonds with residues F153 (1: 2.26 Å), N159 (2: 2.14 Å), and T161 (3: 2.50 Å). Chitin is proposed to form four hydrogen bonds with N159 (1: 2.11 Å), N165 (2: 2.68 Å; 3: 2.37 Å), and S163 (4: 2.18 Å). Mannan is predicted to bind through three hydrogen bonds involving F153 (1: 2.09 Å), V160 (2: 2.17 Å), and L164 (3: 1.98 Å). Hydrogen bonds are shown as cyan lines, and the interacting residues are labeled in distinct colors. (C) Overview of the docking interactions between PxECM33 and β-glucan, chitin, and mannan.

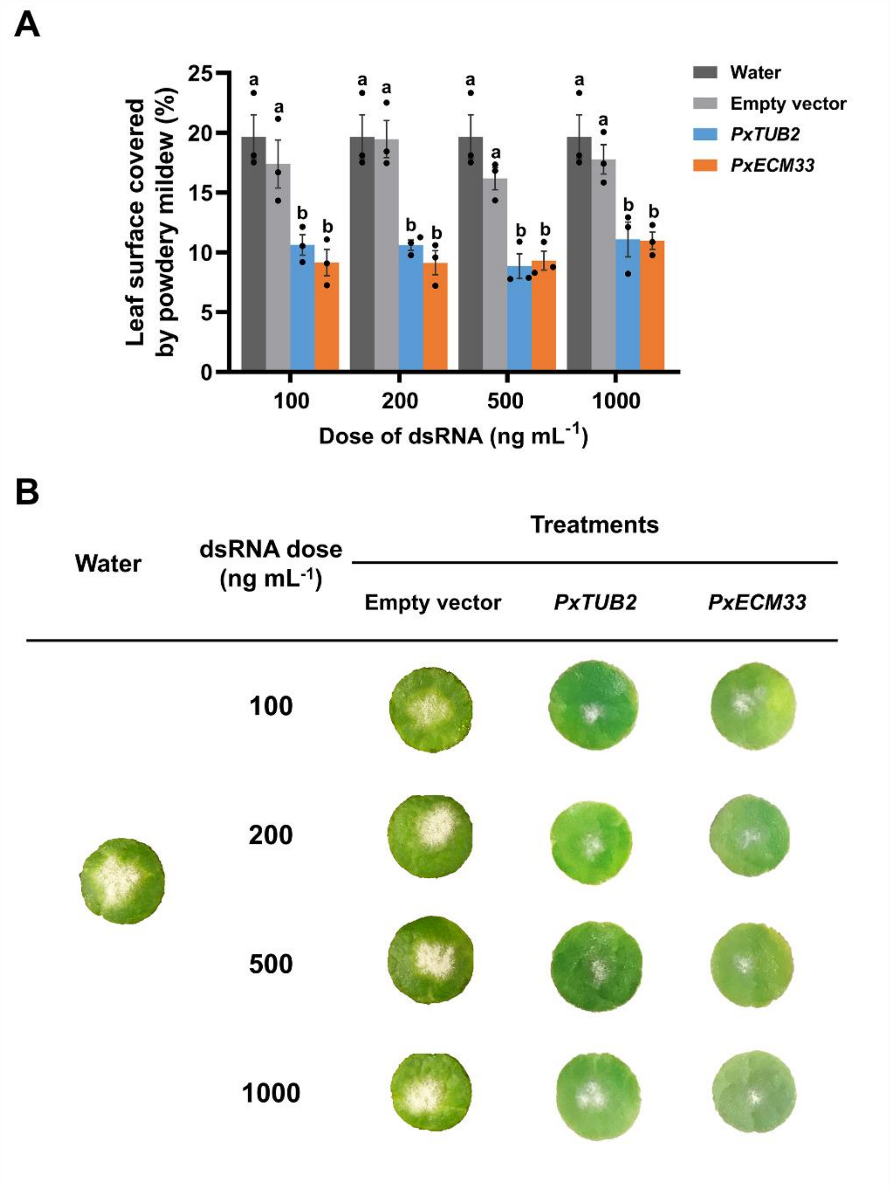

Fig. S4. Effects of exogenous dsRNA application on *P. xanthii* growth and disease development. Melon cotyledon discs were treated with varying concentrations of dsRNA targeting the *PxTUB2* and *PxECM33* genes. After drying, the discs were inoculated with a *P. xanthii* suspension (1 × 10^5 conidia mL^{-1}). Discs treated with water or dsRNA derived from an empty vector served as negative controls. (A) Graph showing the effect of dsRNA treatment on *P. xanthii* growth. Disease severity, expressed as the percentage of the leaf disc surface covered by powdery mildew, was assessed 8 days post-inoculation (dpi). Each data point represents the mean of 36 samples from three independent experiments, with error bars indicating the standard error of the mean. Data points labeled with the same letter are not significantly different ($p = 0.05$, Fisher's LSD test). (B) Representative images of melon cotyledon discs from the assay described in (A), captured at 8 dpi.

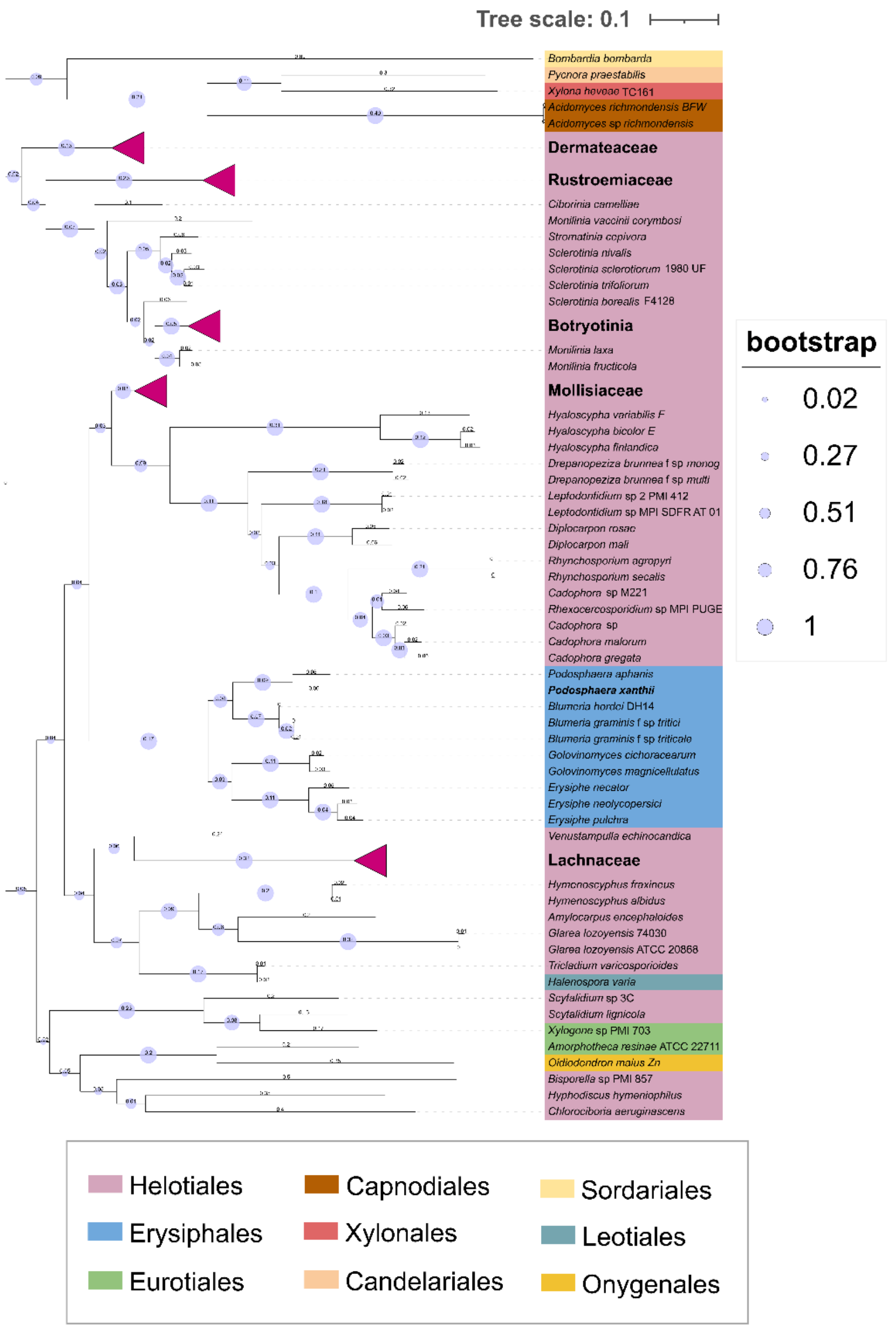

Fig. S5. Phylogenetic analysis of ECM33. Phylogenetic distribution of *PxECM33* homologs across Ascomycota. Protein sequences were retrieved from NCBI using BLASTp. Accession numbers are listed in Table S3. The top 100 amino acid sequences were aligned using MUSCLE, and a phylogenetic tree was constructed with MEGA12 software. Bootstrap values are shown on each branch; values below 0.5 are not displayed. The tree was visualized using the iTOL web server. Erysiphales (powdery mildew fungi) are labeled in blue.

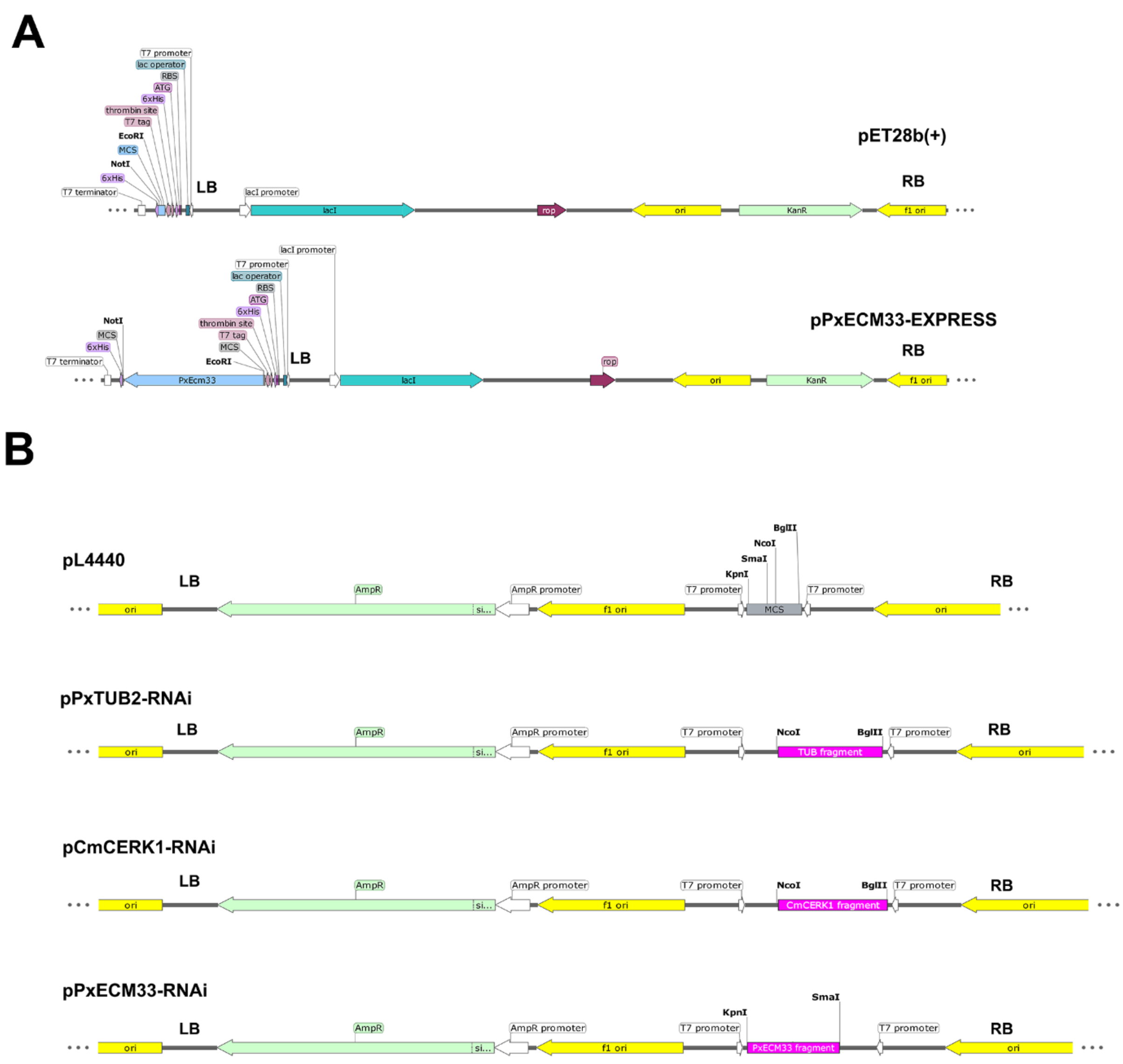

Fig. S6. Schematic representation of the plasmids used in this study. The plasmid maps highlight key features relevant to protein expression or dsRNA synthesis. (A) The coding sequence of PxECM33, lacking the signal peptide (SP) and GPI-anchor sequence, was cloned into the expression vector pET28b(+). Key elements include: LB and RB (left and right borders); T7 terminator (transcription terminator of bacteriophage T7 RNA polymerase); 6×His tag (affinity tag); T7 tag (epitope tag from T7 major capsid protein); thrombin site (recognition and cleavage site); ATG (start codon); RBS (ribosome binding site); lac operator (binding site for the lac repressor, inducible by IPTG or lactose); lacI promoter and lacI gene; rop (repressor of primer); KanR (kanamycin resistance marker). NotI and EcoRI were used to clone the PxECM33 insert. (B) DNA regions of the pL4440 vector used for dsRNA synthesis. Target gene fragments (pink bars) were cloned into the multicloning site (MCS). Key features include: LB and RB; transcriptional direction (arrows); ori (ColE1/pMB1/pBR322/pUC origin of replication); f1 ori (f1 phage origin); AmpR (ampicillin resistance marker) and its promoter; T7 promoters flanking the MCS; and restriction sites (KpnI, SmaI, NcoI, BglII) used for cloning. Pink bars represent the gene fragments used for dsRNA synthesis of *PxTUB2*, *CmCERK1*, and *PxECM33*.

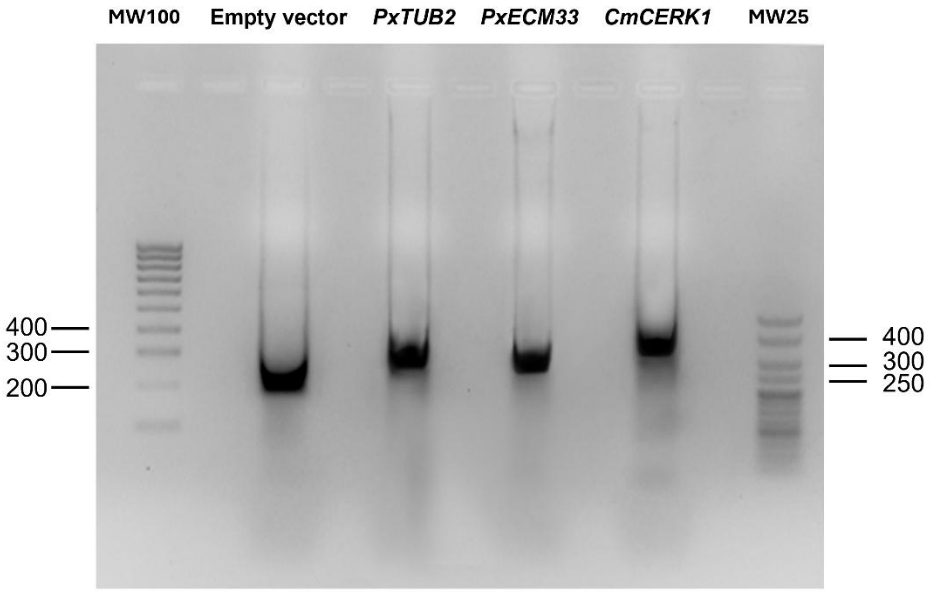

Fig. S7. Integrity analysis of dsRNA products used in this study. dsRNAs were synthesized *in vitro* using the corresponding RNAi silencing vectors, as described in the Experimental Procedures, and their integrity was evaluated by electrophoresis on a 2 % agarose gel. The gel shows dsRNAs targeting the fungal genes *PxTUB2* and *PxECM33*, as well as the melon gene *CmCERK1*. A nonspecific dsRNA containing multiple cloning-site sequences derived from an empty pL4440 vector is also included. Molecular weight markers MW100 (left) and MW25 (right) correspond to HyperLadder 100 bp and HyperLadder 25 bp (Bioline, Meridian Bioscience, US), respectively.
